# Bidirectional interactions facilitate the integration of a robot into a shoal of zebrafish *Danio rerio*

**DOI:** 10.1371/journal.pone.0220559

**Published:** 2019-08-20

**Authors:** Vaios Papaspyros, Frank Bonnet, Bertrand Collignon, Francesco Mondada

**Affiliations:** 1 Biorobotics Laboratory, Institute of Bioengineering, École Polytechnique Fédérale de Lausanne (EPFL), Lausanne, Switzerland; 2 Unit of Social Ecology (USE), Université libre de Bruxelles (ULB), Bruxelles, Belgium; Oregon State University, UNITED STATES

## Abstract

Many studies on collective animal behavior seek to identify the individual rules that underlie collective patterns. However, it was not until the recent advancements of micro-electronic and embedded systems that scientists were able to create mixed groups of sensor-rich robots and animals and study collective interactions from the within a bio-hybrid group. In recent work, scientists showed that a robot-controlled lure is capable of influencing the collective decisions of zebrafish *Danio rerio* shoals moving in a ring and a two-room setup. Here, we study a closely related topic, that is, the collective behavior patterns that emerge when different behavioral models are reproduced through the use of a robotic lure. We design a behavioral model that alternates between obeying and disobeying the collective motion decisions in order to become socially accepted by the shoal members. Subsequently, we compare it against two extreme cases: a reactive and an imposing decision model. For this, we use spatial, directional and information theoretic metrics to measure the degree of integration of the robotic agent. We show that our model leads to similar information flow as in freely roaming shoals of zebrafish and exhibits leadership skills more often than the open-loop models. Thus, in order for the robot to achieve higher degrees of integration in the zebrafish shoal, it must, like any other shoal member, be bidirectionally involved in the decision making process. These findings provide insight on the ability to form mixed societies of animals and robots and yield promising results on the degree to which a robot can influence the collective decision making.

## Introduction

From the middle of the twentieth century, modelling collective behavior has been in the center of attention of many biological, physical, and computational studies. The main motivation behind such studies lies in the rather complex behaviors that can arise from simple rules on the individual level [1]. Such phenomena are quite common in nature and range from the molecular level, to the subject of this study, collective animal behavior. Disentangling the dynamics of such collective phenomena can reveal information concerning the underlying structure and organization of multiple physical phenomena observed in nature [2]. However, modelling these phenomena requires rigorous data analysis to extract formulation that can reproduce, at least in theory, the observed collective dynamics. In collective motion of fish, the focus of this paper, there exist various models describing their potential interactions rules [3–16]. More often than not, to compare the quality of different potential models, scientists have evaluated their likelihood to reproduce or predict experimental data [17, 18]. However, this approach does not guarantee any success since different sets of rules are able to reproduce the observed collective movements. One way to overcome these limitations is to test models in real conditions. However, their implementation and validation in physical, real-time and noisy systems is a technical challenge that was only recently solved thanks to the development of micro-electronic and embedded systems. Therefore, in the past years, robots and artificial lures allowed scientists to put these theoretical models to the test in real-life scenarios and with true feedback from the animals, in order to study their collective behavior. Thus, they have since been increasingly involved in inferring the rules of interaction among animals such as bees [19–21], fish [22–27], birds [28–30] and cockroaches [31].

Some of them [32–34] relied on the use of teleoperated devices that produce signals (e.g., visual, acoustic, electric) to attract or repel the animals, others rely on mobile robots that are not explicitly mimicking the animal under study (e.g., it could be a sheepdog among sheep [35]) and some relied on mimetic lures, that is, on lures that mimic the shape, size, and appearance or behavior [22, 23, 31, 36, 37]. These studies demonstrated that artificial agents able to perceive and emit pertinent and adapted signals can influence and control self-organized choices by mixed groups of animals and robots [38].

Although robots proved to be a valuable asset in this context, they also uncovered the need for behavioral models that are applicable to robotic platforms. As biological systems are characterized by great variability and diversity of interaction dynamics, it is very challenging to evaluate the degree to which a robot or robotic lure is integrated within it. A first strategy that addresses this issue relies on open-loop models [39–54] that do not actively react to or take into account the actions of the animals. In this case, it may be difficult to discern if the focal animal is reacting to external stimuli or to another agent considered as a shoal member. Therefore, they may often fail to provide a clear insight into the internal decision making process and the natural information flow between the shoal members. A second strategy is based on closed-loop models [23–26, 55–58] attempting to achieve a conspecific status among individuals by engaging in mutual information exchange and could reveal the intrinsic decision making mechanism of individuals. In [55], the authors compared the success of a robotic lure to integrate among a shoal of zebrafish *Danio rerio* by increasing the biomemitic characteristics of the artificial agent (biomimetic or non-biomimetic lure, movement pattern, and trajectory) and showed that the level of integration increases with the biomimetism of the robotic agent. While similar studies have been conducted with the use of computer animations [59], none of the former studies explicitly compared behavioral models of interaction to evaluate their relative performance when a physical device (i.e., a robot) is used for the interaction.

Therefore, the following research question arises: can we discriminate between different behavioral rules by implementing them in a robot interacting with a shoal of zebrafish? Here, we studied this research question as follows: (1) we make use of a circular corridor setup which serves as a baseline control experiment arena for numerous studies on collective behavior [27, 60–62]; (2) we designed three behavioral models exhibiting three different dynamics, namely, a purely reactive model that explicitly follows the fish, an imposing direction model constantly attempting to dictate the collective swimming direction decision and a biomimetic model mimicking the decisions of zebrafish in a circular corridor environment; (3) we performed experiments with zebrafish-only and mixed groups of robots and zebrafish; (4) we analyzed the results using three inherently different approaches. We show that our robotic system was capable of participating in the collective decision making and blending in the shoal without perturbing its interaction dynamics. This, in turn, led to an improved integration state where the robot was not only accepted by the shoal, but it was also contributing to the collective decision and acting as a leader for the majority of the time.

## Materials and methods

### Animals

The authorization for the experiments conducted in this research work was approved by the state ethical board of the Department of Consumer and Veterinary Affairs of the Canton de Vaud (SCAV) of Switzerland (authorization № 2778).

For the experiments, 60 wild-type zebrafish *Danio rerio* with short fins were used (AB strain). The zebrafish were acquired from a pet shop and subsequently stored in a 60-litre aquarium. The average length of the zebrafish used was approximately 4 cm. Water in the housing aquarium was kept at a temperature of 26°C. The fish were fed once per day with commercial food between 16:00 and 18:00. Furthermore, the aquarium was enriched with plastic plants, Cladophora, gravel, rocks, and aquatic snails.

### Experimental setup

**Fig 1:**
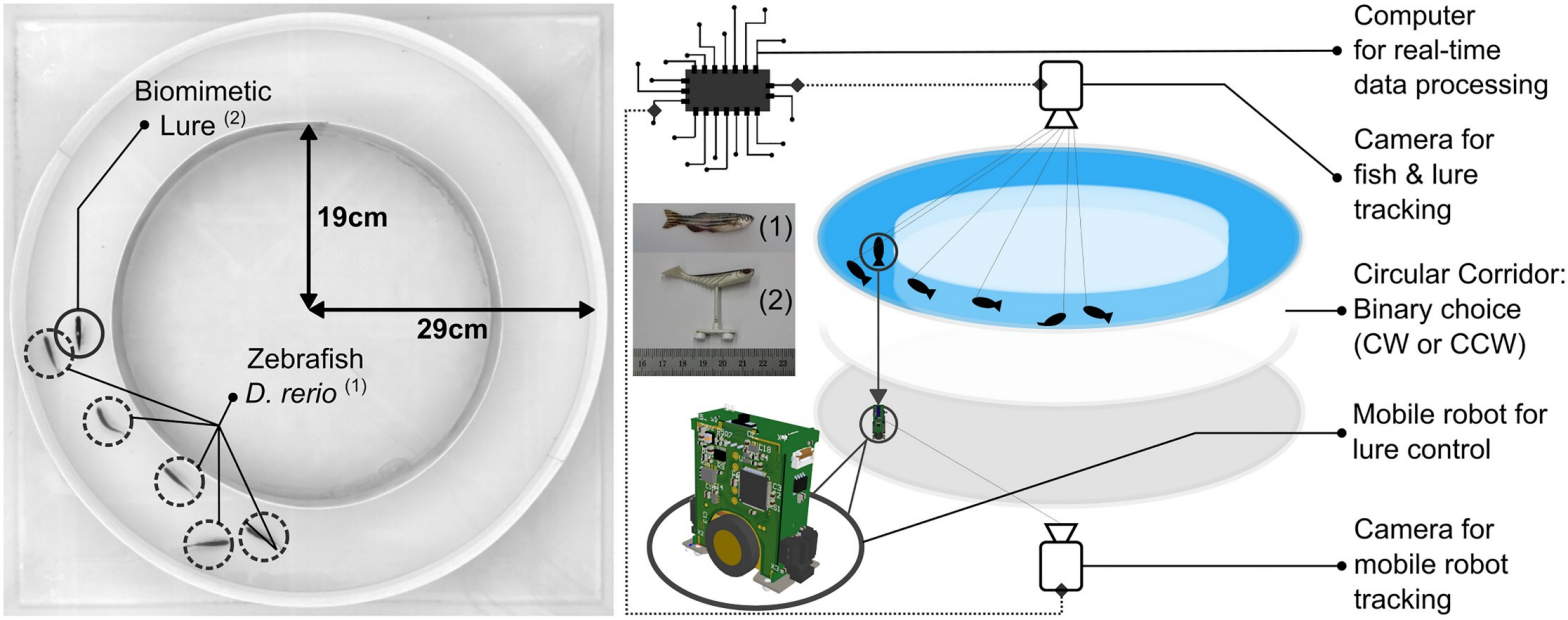
Experimental setup. (left) Top view depicting the setup’s dimensions, i.e. inner ring radius of 19 cm and outer of 29 cm. The dotted circles indicate the positions of zebrafish while the full circle indicates the position of the biomimetic lure, and (right) breakdown view of the setup depicting individual components that are necessary for closed-loop interaction. The mobile robot (FishBot) is moving below the tank and drives a biomimetic lure inside the tank through a magnetic coupling. The top and bottom mounted cameras capture frames at a rate of 15 Hz and transmit the information to a computer. The computer will then fuse the information to determine the positions and heading of fish and robot(s) alike. In gray, we denote the conductive plates that are used to power the FishBot.

The experimental arena pictured in Fig 1 consists of a 10 cm wide circular corridor (from two circular walls: an outer of 58 cm diameter and (2) an inner of 38 cm diameter) placed in a 100 × 100 × 25 cm^3^ glass tank, as in [27]. This setup presented the zebrafish with a binary choice for movement: i.e they could either move clockwise (CW) or counter-clockwise (CCW). In fact, this is a common setup for behavioral studies [27, 61–63] because it allows for setting aside spatial complexities and instead provides a symmetric arena that enables researchers to analyze multiple instances of the same behavioral traits (e.g., U-turns [60] where the fish will perform a direction change greater than or equal to 180°) and quantify their consistency across different types of behavioral models.

The bottom part of the experimental tank was covered with a Teflon plate to allow for smoother motion of the robotic fish lure (see Robotic system for closed-loop zebrafish-robot interactions) and avoid any stimuli produced by reflections or by the mobile robot moving below the setup. Furthermore, the setup was confined behind white sheets to isolate the fish from the rest of the room, while also maintaining a consistent lighting environment. A uniform luminosity for the room was provided by four 110-W fluorescent lamps placed at each of the four sides of the tank.

### Robotic system for closed-loop zebrafish-robot interactions

**Fig 2:**
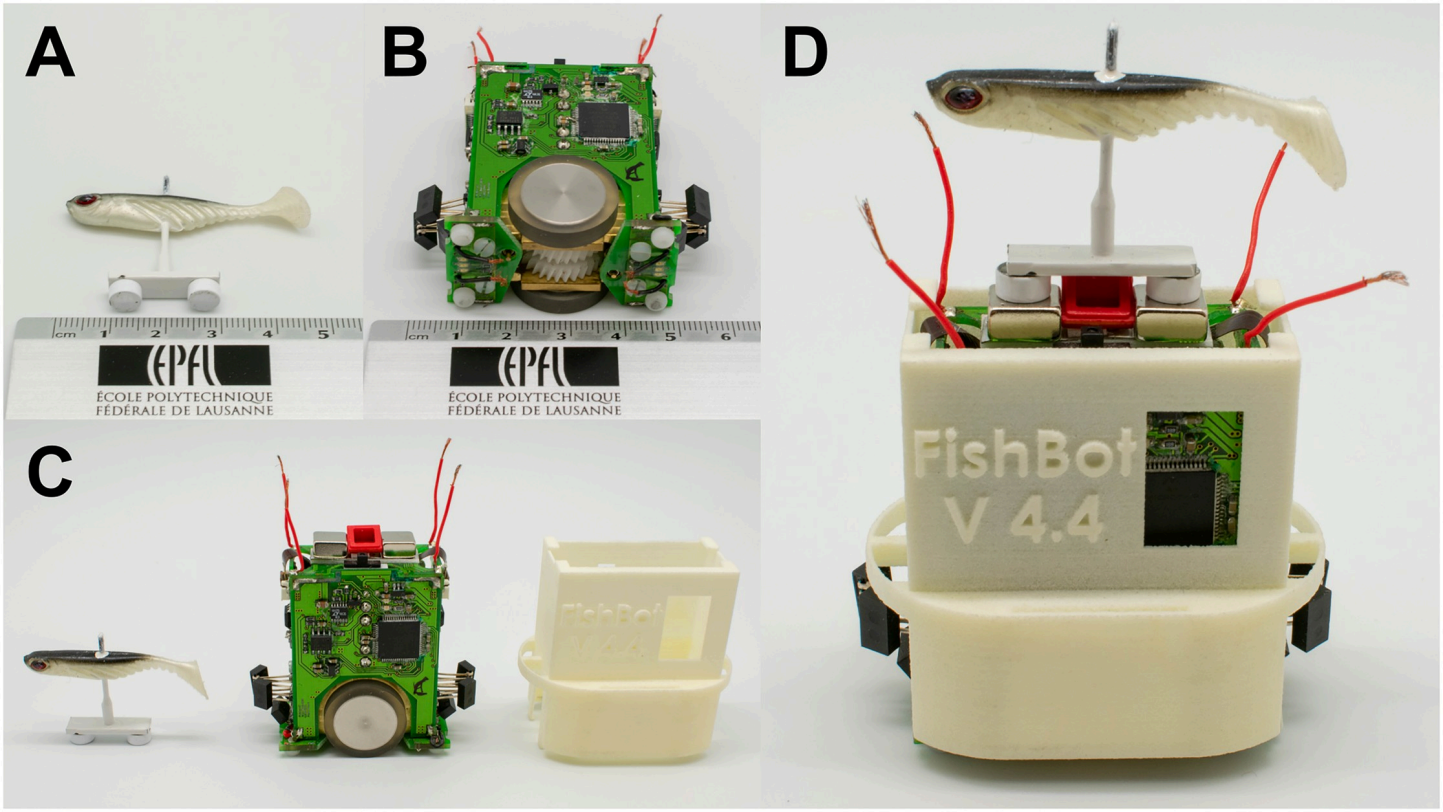
FishBot and biomimetic lure. (A) The biomimetic lure (approximately 4.5 cm long) fixed on a white carbon stick, (B) the FishBot on its side (approximately 5.5 cm long), (C) relative size of the lure, FishBot and FishBot cover (from left to right), and (D) assembled robotic system; the white cover protects critical parts of the FishBot, and the lure magnetically coupled to the FishBot.

For the zebrafish-robot interaction experiments we used one miniature wheeled robot, the FishBot [22, 64] (see Fig 2B). The robot was placed between two conductive plates located below the experimental setup (see Fig 1) and was powered using brushes that were constantly in contact with them. This configuration allowed the robot to operate for long periods of time and powered the motors that were, in turn, capable of achieving the necessary speed and acceleration in order to quickly adapt to the rapid spatial displacements of zebrafish. The FishBot was additionally equipped with a Bluetooth chip that allowed it to wirelessly communicate with a computer that was providing the necessary motor commands.

A soft biomimetic lure of approximately 4.5 cm length (see Fig 2A) was selected to physically interact with the animals. This lure was designed to mimic the morphology of the zebrafish and passively beat its tail during its underwater motion. As described in [22] this specific lure achieved strong acceptance in groups of zebrafish. Subsequently, it was mounted on a carbon stick at a height of 3 cm to ensure that it was visible by neighboring fish. An iron plate located at the bottom of this stick carried two magnets that allowed for a magnetic coupling (similarly to [22–26], see Fig 2D) to the robot located below the setup.

### Control and tracking software

In order to close the interaction loop between the fish and the robot we made use of the Control and tracking for multi-agent animal-robots groups (CATS) framework [65]. CATS continuously monitored the positions of the robot and the animals through image frames obtained by the two cameras located above and below the setup (see Fig 1). In particular, the overhead camera was set to simultaneously stream video in two resolutions; (1) a 1040 × 1040 stream that was recorded and used for the analysis of the experiment, and (2) a 512 × 512 stream that was used in CATS for the detection of the fish and/or lure in real-time. The image frames from the camera located at the bottom of the setup were directly fed to CATS for processing.

More specifically, the agents’ positions were determined by feeding the image frames of the overhead camera to a corner detection method [66] implemented using the OpenCV [67] library; while the camera located below the setup was used to localize the mobile robot, which was equipped with 6 light-emitting diodes of blue color, using a blob detector. Subsequently, CATS fused information from both cameras to distinguish the artificial lure from the living individuals. We note that both cameras operated at a rate of 15 frames per second.

Once CATS had finished determining the positions, the resulting spatial information (2D position and heading direction) became available in the control layer of CATS. This layer is responsible for the higher level control procedure of the robot (i.e., deciding which is the next desired state for the robot). The behavioral models presented in the following section were implemented within the control layer of CATS and output higher level commands such as desired velocity, position, and orientation. Those commands were then fed to a micro-controller unit where a proportional-integral-derivative controller (PID) translated the higher level commands to motor commands, similarly to [64].

Additionally, to the online control procedure that was devised for the fish-robot interaction experiments, the videos of each experiment underwent post-processing using the idTracker software [68] to extract the trajectories of each agent for each experiment. This time-consuming and computationally expensive process is capable of recreating the trajectories of each identified agent (6 agents for 30 minute long experiments) with on average 95% accuracy, correcting any mistakes made in trajectories due to crossings that occurred naturally throughout the experiment.

### Experimental procedure

For the duration of the experiments, we maintained a constant height of 6 cm of water in the setup. Under these conditions, the fish were not additionally stressed, and their movement was on average constrained to a specific height, thus, reducing spatial complexities on the z-axis. Prior to placing the zebrafish in the setup, the water temperature was brought to 26°C. Thereafter, a shoal of zebrafish was randomly caught from the rearing tanks with a fishnet and placed in the experimental setup. After a 5 minute acclimatization period during which the FishBot remained stationary, we started and recorded the experiment for 30 minutes. No individual was used twice in the same day.

We conducted 10 experiments with shoals of six zebrafish and no FishBot to observe the baseline behavior of the fish (hereby referred to as “fish-only” experiments) when no artificial stimuli were provided. Then we conducted a total of 3 × 10 experiments with five zebrafish and one FishBot with three different behavioral models (described in the following section) for the FishBot. Each model was tested in random order to account for the fish getting accustomed to a specific behavior exhibited by the robot.

### Behavioral models

#### Follower model (FM)

We designed a closed-loop following model, the “Follower Model”, where the robot was simply instructed to head towards the point in space that was on average most dense in terms of fish occupancy. FM is a purely reactive, passive model in the sense that it does not actively model or embed interaction in its design and instead reacts only to the position of the fish by always following it.

#### Despotic model (DM)

We also designed an open-loop model, which is an adaptation of the approach described in [27] that uses only one robot which was instructed to perform a CW movement throughout the experiment. Contrary to FM, this model is always attempting to impose the collective movement direction decision, thus we call it “Despotic Model”.

#### Feedback-Initiative model (FIM)

Finally, we implemented a closed-loop parametric behavioral model similar to [61]. In [61], the authors described a model that operates in a one-dimensional decision space (i.e., CW or CCW movement). First, the circular corridor arena is divided into 40 equal cells of 9-degree arc length each. Then, the focal individual will take a directional decision according to the perceived heading directions of its neighbors and a probability to “disobey” this collective decision. More specifically, the next heading direction of an individual is given by the following expression:

$$h\left({\text{fish}}_{j},\;t+1\right)=\;\frac{h\left({\text{fish}}_{j},\;t\right)\;+\;\sum_{i=0,\;\;i\neq j}^{N_{P}}h\left({\text{fish}}_{i},\;t\right)}{\left|h\left({\text{fish}}_{j},\;t\right)\;+\;\sum_{i=0,\;\;i\neq j}^{N_{P}}h\left({\text{fish}}_{i},\;t\right)\right|},$$ (1)

where *h*(fish_*j*_, *t*) ∈ {−1, 1} the heading direction at time *t* and *N*_*P*_ the subset of the set of all individuals, *N*_*A*_, that are in the perceptual range of the focal individual. The perceptual range is defined as the set *F* ⊆ *N*_*A*_ of the individuals that are within *p*_*r*_ cells in the forward direction of the focal individual. At every time-step and after computing the new heading direction, the focal individual is given a probability 1 − *P*_obey_ to choose the opposite direction to the one computed through the above interaction metric. We refer to this opposition to the collective decision of swimming direction as the initiative of the focal individual.

**Fig 3:**
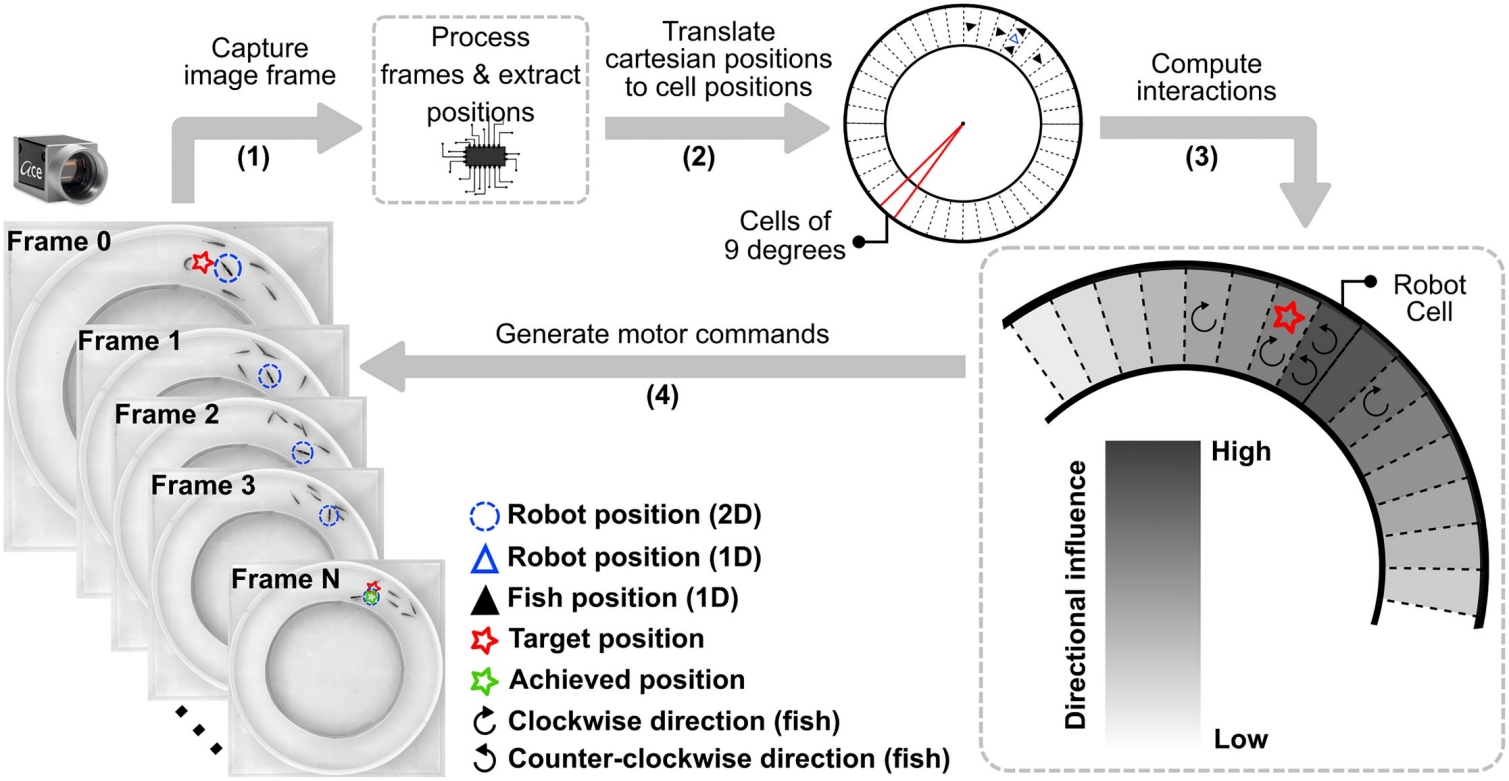
Closed-loop robot control. For each system cycle: (1) a high resolution image frame is captured by the overhead camera, (2) the frame sent to a high performance computer, where it is processed to determine the positions, velocities, and headings of each individual, (3) the extracted positions are discretized and each individual is placed in its corresponding cell and (4) the discretized positions and headings of each individual are forwarded to the FIM, which in turn, weighs the heading direction of the neighboring individuals and produces a desired position (red star) for the next timestep. After a few time-steps an approximation of the target position will be achieved (green star) and the process is repeated.

In order to investigate the degree to which our robot can influence the directional decisions taken by the group, we propose a variant of this model where the focal individual attempts to closely mimic zebrafish interaction in similar arenas [27, 61, 63] while at the same time it intelligently embeds initiative in its decision making according to the feedback perceived by the neighboring social companions. Therefore, we call this model “Feedback-Initiative Model”. Similarly to [61], we discretized the circular corridor in cells and controlled the robot’s direction of movement in the one-dimensional space of CW or CCW movement. More specifically, we separated the setup in 40 cells each of which corresponds to approximately 4 cm of arc length, the average length of a single zebrafish. Essentially, increasing the number of cells, i.e., decreasing the arc length per cell, would allow for more detailed separation of the fish in terms of cell occupancy, but would be subject to the noise produced by CATS (see Control and tracking software). This discretization process reduced the locomotion control dimensions to 1D space and the model need only to output a simple instruction at every time-step: move one cell CW or CCW. The model made a prediction about the best candidate direction at time *t* + 1, every 0.25 seconds (i.e., the controller time-step is equal to 0.25 seconds). In the context of this study, the best candidate swimming direction was considered to be the one that has the highest probability to elicit a collective U-turn (i.e., a switch of the swimming direction for the majority of individuals). A complete system cycle of the model is depicted in Fig 3.

Our source of inspiration for those interactions was derived by the innate behavior of zebrafish in similar setups and can be summarized in the following two key features for a focal individual: (1) an innate tendency to align with the shoal and (2) a tendency to perform a U-turn when few or no agents are in the field of view of the focal, initiating a direction change that might propagate throughout the shoal [3, 62, 63, 69]. The former is formalized as a weighted sum of the direction of a focal individual’s neighbors and is defined as the dot product of the heading (-1 or 1) and an exponential function that can rapidly increase or decrease the impact of a neighbor according to its position, as follows:

$$\begin{array}{c} h\left({\text{fish}}_{j},\;t+1\right)=\sum_{i=0,\;i\neq j}^{N_{A}}h\left({\text{fish}}_{i},\;t\right)\cdot e^{\alpha \cdot p({\text{fish}}_{i},\;t)} \end{array}$$ (2)

where $p\left({\text{fish}}_{j},\;t\right)\in \left[0,\right|N_{C}\left|\right)\subseteq Z$ the position of the fish *j* at time *t*, *N*_*C*_ the set of cells that correspond to the discretization process and a∈R a regulatory parameter to set the slope.

To allow for more flexibility in FIM we used two separate parameters *α*_*f*_ and *α*_*b*_ for fish that are in leading and following positions respectively (see Algo. 1) and defined the sets *F* and *B* of fish in the forward and backward positions respectively. Intuitively, the difference between the parameters *a*_*f*_ and *a*_*b*_ can express biologically observed behaviors such as that fish within the immediate field of view of a focal individual have more influence on it [3, 60, 62, 69], while the followers can still be perceived due to the water flow [70] and might have less, but significant influence. The parameters *α*_*f*_ and *α*_*b*_ were manually tuned for this specific configuration and are given the values -0.2 and -0.5, respectively. Despite the sum’s ability (see Eq 2) to phase out the influence of perceived agents over distance, we explicitly limited the robot’s knowledge within 15 cells (*p*_*f*_ = 7 cells forward, *p*_*b*_ = 7 cells backwards and current cell occupied; or 135 degrees of perceptual range for the choice of 40 cells). Indeed, for very low values of *α* (see Eq 2), a conspecific could have been perceived in front and behind the focal fish due to the circular design of the setup. Finally, the focal individual’s tendency to perform a U-turn and even disobey the collective decision of the shoal concerning the direction of movement was modeled as a probability *P*_*obey*_. The probability *P*_*obey*_ is dependent on the amount of fish in the forward direction to account for an individual’s intuition to not wander too far from the fish school or to simply initiate a random direction change. More specifically, this probability is regulated by two constant parameters (see Algo. 1): (1) *a*_influence_ = 4, which allows one to increase or decrease the amount of influence of forward individuals concerning the obedience (2) a constant upper bound *τ* = 0.95, value which we estimated through past fish-only experiments. A subset of the parameter space that could be used in different scenarios include:

- *α*_*f*_ = *α*_*b*_ = −inf, *τ* = 1 ⇒ *P*_obey_ = 1, will produce purely following behavior.
- *α*_*f*_ = *α*_*b*_ = *τ* = 0 ⇒ *P*_obey_ = 0, will produce a behavior that always contradicts the collective.
- *a*_influence_ = 0, will retain a constant and neighbor-independent *P*_*obey*_.
- Setting the perceptual range to zero (i.e., *Fish*′ = ∅) and *τ* = 1, will produce an imposing direction behavior (the initial direction will be followed throughout the experiment).

We designed this model to be parametric and include stochastic elements of decision making. The parametric design allows for modification of the model to comply with different scenarios of interaction or species of fish (e.g., the robot could be instructed to emphasize on following by changing a few parameters), either prior to deployment or during an experiment, while the stochasticity serves as a way to promote initiative in the model. In [61] the authors described a model where the focal individual’s next direction will be with high certainty decided by the average swimming direction of the neighboring individuals. Conversely, here the goal was to elicit a different effect from the fish and influence them to change their swimming direction.

**Algorithm 1**

 **procedure** Stimulate(Fish′)

   Split Fish′ to:

    *C* = {fish in focal cell} ⊆ Fish′

    *F* = {fish in *p_f_* forward cells} ⊆ Fish′

    *F* = {fish in *p_b_* forward cells} ⊆ Fish′

 $s=\sum_{i=0,\;\;{\text{fish}}_{i}\in \;F}^{\left|F\right|}h\left({\text{fish}}_{i},\;t\right)\cdot e^{\alpha_{f}p\left({\text{fish}}_{i},\;\;t\right)}+\sum_{j=0,\;\;{\text{fish}}_{j}\in \;C\cup \;B}^{\left|C\cup \;B\right|}h\left({\text{fish}}_{i},\;t\right)\cdot e^{\alpha_{b}\cdot p\left({\text{fish}}_{j},\;\;t\right)}$

 $h\prime =\{\begin{array}{lll} s & , & s\neq 0\\ h\left(\text{focal},\text{t}\right) & , & otherwise \end{array}$

 $P_{\text{obey}}=\;\{\begin{array}{lll} \tau *(1-{\left(\left|F\cup C\right|+1\right)}^{-\alpha_{\text{influence}}}) & , & \text{Fish}\prime \neq \emptyset \\ \tau & , & otherwise \end{array}$

  with probability 1 − *P*_obey_ reverse *h*′

  **return** *h*′

 **procedure** Move(*h*(focal, *t* + 1)

  *p*(focal, *t* + 1) = *p*(focal, *t*) + (*h*(focal, *t* + 1)

 **procedure** FeedbackInitiativeModel

  ∀*fish* ∈ Fish, where |Fish| = |*N*_*A*_|:

   Initialize **position** ∈ [0, |*N*_*C*_|)

   Initialize **heading** ∈ {CW = −1, CCW = 1}

   **while** stopping criteria not met **do**

   ∀*fish* ∈ Fish:

     Fish′ = {fish within the perceptual range of the focal fish} ⊆ Fish ∪ ∅

     *h*(focal, *t* + 1) = Stimulate(Fish′)

     Move (*h*(focal, *t* + 1))

### Data filtering

The 15 frames per second capture rate made it possible to detect even minor fluctuations in the displacement of an individual. On one hand, this rate is useful for tracking fast moving objects or animals, but, in the case of zebrafish that move with an average speed below 20-25 cm/second in this setup, it might induce noise due to temporary loss of the position or the image processing algorithm reporting minor differences in the position of an individual at every time-step. Therefore, throughout the following experiments we filtered the data reported in two ways: (1) 3 frames (0.2 seconds of interaction) were averaged to calculate the centroidal position, heading or velocity of each agent and (2) the behavioral models presented in following sections discretized the positions in bins, the number of which was selected to further filter the measurements where necessary.

### Data analysis

In this section, we introduce a set of metrics based on spatial, directional and information theoretic measures, as well as the statistical methods followed to evaluate and compare the behavioral models. We note that all the raw trajectory data are available at https://github.com/epfl-mobots/plos_one_experiments.

#### Average angular distance

In collective behavior, denser groups often suggest a more cohesive, aligned and organized movement [63, 71], thus, density-based measures have been widely used for inferring the interaction rules within a group of animals [72, 73]. Here, we computed a similar measure, by calculating the average angular distance between all pairs of agents. The angular distance between two fish is defined by the angle *θ*_*ij*_(*t*) at time *t*, where *i*, *j* are two individuals and *θ*_*ij*_(*t*) ∈ [0, *π*] is the angle between *i* and *j* with respect to the origin point of the setup (center of both rings). We note that *θ*_*ij*_(*t*) refers to the acute angle between the two individuals (i.e., we only evaluate the angular proximity). The average angular distance was computed as the average of all the pairwise angular distances and is summarized in the following expression:

$$\begin{array}{c} \text{averageAngularDistance}\left(t\right)=\frac{1}{N_{A}\left(N_{A}-1\right)}\sum_{i=1}^{N_{A}}\sum_{j=1,\;i\neq j}^{N_{A}}\theta_{ij}\left(t\right) \end{array}$$ (3)

#### Collective U-turns

Although the average angular distance provides useful topological information concerning the closeness of the group and thus its cohesive and synchronized movement, it would be incomplete without a complementary metric concerning the interactions within it. Here, we captured these interactions in the number of collective direction changes performed (e.g., from CW to CCW or vice versa), which in this binary choice scenario we defined as collective U-turns. The U-turn in schools of fish has attracted attention [60, 62, 63] as it provides insight on how information is propagated among individuals. Consequently, the effect that each behavioral model has on the occurrence of collective U-turns is representative of its ability to mimic, modulate or perturb the collective decision making.

To calculate the number of collective U-turn events we first defined the polarization of a zebrafish shoal in this context, as follows:

$$\begin{array}{c} \text{pol}\left(t\right)=\frac{1}{N_{A}}\sum_{i=0}^{N_{A}}h\left({\text{fish}}_{i},\;t\right) \end{array}$$ (4)

A collective U-turn occurs when the polarization of the shoal switches from one direction to another (pol(*t*) ⋅ pol(*t* − 1)<0; i.e., we did not take into account transitions from CW/CCW to 0). Complementary figures concerning the duration of consecutive movement before a collective U-turn occurs are available in the Supporting information. section (S1, S2, S3, S4, S5, S6, S7 and S8 Figs).

#### Transfer entropy

To complete the aforementioned metrics we also employed an information theoretic measure based on the Shannon entropy [74], called transfer entropy (TE) [75, 76]. Recent studies in collective behavior [43, 60, 77–80] have been increasingly using TE to provide insight on the mutual interactions of individuals over time or with time delays [81]. Here, we adopted the notation of TE with embedded delay, as defined in [82]. More specifically, given two time-series *X* and *Y*, TE measures the amount of information provided by the source *X* about the target *Y* and is defined as follows:

$$\begin{array}{c} T_{X\to Y}=\sum p\left(y_{n+1},y_{n}^{\left(k\right)},x_{n}^{\left(l\right)}\right)\log\frac{p\left(y_{n+1}\right|y_{n}^{\left(k\right)},x_{n}^{\left(l\right)})}{p\left(y_{n+1}\right|x_{n}^{\left(k\right)})} \end{array}$$ (5)

where *l* and *k* are the history lengths for the two time-series:

$$\begin{array}{c} x_{n}^{\left(l\right)}=\{x_{n},\;x_{n-\tau_{k}},\;x_{n-2\tau_{k}},\ldots ,x_{n-\left(k-1\right)\cdot \tau_{k}}\} \end{array}$$ (6)

$$\begin{array}{c} y_{n}^{\left(l\right)}=\{y_{n},\;y_{n-\tau_{l}},\;y_{n-2\tau_{l}},\ldots ,y_{n-\left(l-1\right)\cdot \tau_{l}}\} \end{array}$$ (7)

and *τ*_*k*_, *τ*_*l*_ the time delay for the source and destination signal respectively.

In the context of this research work, we defined the time-series *X*, *Y* to be the direction of two separate individuals over time. More specifically, we represented the direction of each agent in a discrete signal with values -1 (CW) or 1 (CCW), by sampling the direction of each individual every 0.2 seconds. For this computation, a more detailed trajectory is required and thus we used a discretization with a cell count of *N*_*C*_ = 160 (i.e., 1 cm per cell; for this procedure, we used the positions extracted from idTracker). In case an individual had not moved during this period, we assumed that its heading has remained the same as in the previous time-step. Furthermore, considering that the influence of one individual to another will be delayed in time, we shifted the source and target time-series by a factor *τ*_*k*_ and *τ*_*l*_, respectively. The intuition behind this measure is that given the direction *Y*_*n*_ of an individual, we gain information about the next direction *X*_*n*_ of another individual. It is rather obvious that the direction change of single individual will not propagate instantly and, thus, there exist the parameters *τ*_*k*_ and *τ*_*l*_ that express this delay.

However, choosing the latter parameters is a non-trivial task, as the values need be meaningful with respect to the experiment in question and at the same time expressive enough to allow for observing potential differences in the fish-robot experiments. To that end, we adopted the same technique of the authors in [60], that is, we run a simple search algorithm to find the parameters that maximize the average TE for the fish-only experiments. To reduce the size of the search space, we only considered values of *k* ∈ [1, 15] and *τ*_*k*_ ∈ [1, 15] (i.e., up to three seconds of signal length and delay). We explicitly set the target delay to *τ*_*l*_ = 1 and the length to *l* = 1 (i.e., 0.2 seconds) as we are interested in the effects of the source signal. We note that the robot-fish experiments were considered for this optimization step to account for the bias that was introduced due to the use of the lure and the models.

Subsequently, we calculated all the pairwise TE values and computed the mean TE across all individuals during one experiment. To do so, we used the JIDT [83] framework to calculate the TE with the optimized parameters *k* = 4 and *τ*_*k*_ = 1 (i.e., *k* = 4 corresponds to 0.8 seconds of history and *τ*_*k*_ = 1 corresponds to a delay of 0.2 seconds). The optimized parameters are empirically found to correspond the time that is necessary for an individual to perform a U-turn and fully propagate it to the shoal.

We computed two separate mean TE values: one for the outgoing and one for the incoming amount of information exchanged. For each case we sum the resulting TE for the trajectories of all the fish-fish or fish-robot pairs. Intuitively, the metric expresses the average information flow direction (incoming or outgoing) when the robot is used. For each behavioral model and each of the outgoing and incoming cases we computed three different quantities: (1) the overall TE for all individuals (2) the TE related to the robot alone (outgoing and incoming) and (3) the average TE of all fish (i.e., excluding the robot’s contribution).

#### Statistical tests

To further validate the interpretation of the resulting data, we performed a Kruskal–Wallis (KW) test followed by a post hoc analysis using Tukey’s honest significant difference (T-HSD), for each measure presented in the following section. The Kruskal–Wallis is chosen due to the fact that the variance of the data-sets in question differs depending on the experiment type.

## Results

### Average angular distance

**Fig 4:**
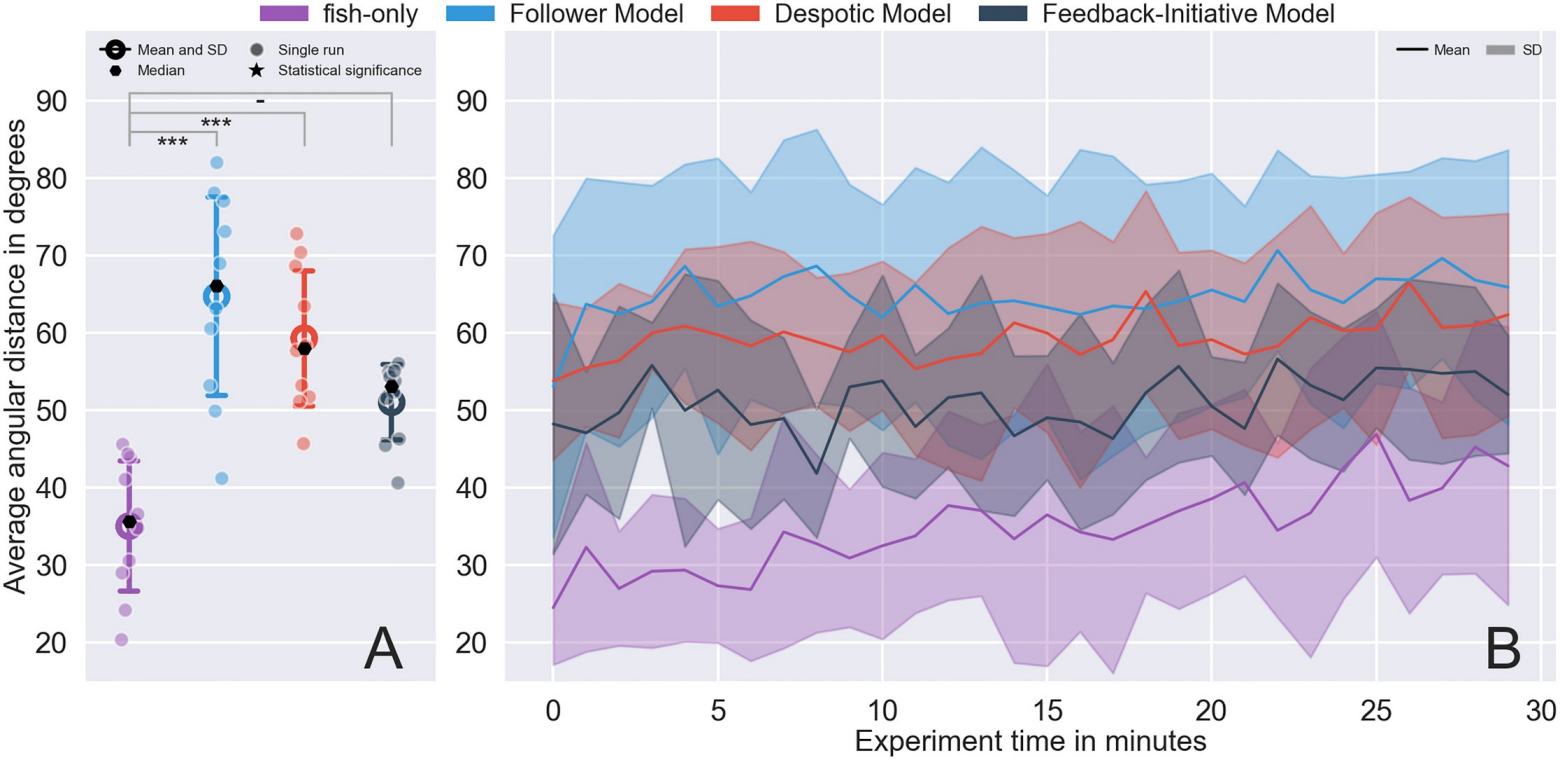
(A) Average angular distance in degrees over ten runs and (B) average angular distance in 1-minute time-steps over all replicates. Annotations of the statistical significance (Kruskal–Wallis test folowed by Tukey’s honest significant difference criterion post hoc analysis) are marked with a dash or stars. The dash corresponds *p* > 0.05, a single star to *p* < 0.05, two stars to *p* < 0.01, three stars to *p* < 0.001. and four stars to *p* < 0.0001.

First, we compiled the average angular distance between the shoal members across the entire observation period for each experiment of the four tested conditions (Fig 4). For the fish-only condition (i.e., where no artificial stimuli are present in the setup), we observed an average angular distance of 35.04 ± 12.62 degrees. Compared to this baseline measure, all models showed a higher inter-individual angular distance (FM: 64.72 ± 15.95 degrees, DM: 59.31 ± 11.87 degrees, FIM: 51.04 ± 10.68 degrees), with these distributions differing significantly from each other (KW test, *p* < 0.0001, *χ*^2^ = 22.45). A more detailed comparison revealed that both the FM and DM significantly differ from the fish-only (T-HSD post hoc test, *p* < 0.0001 and *p* < 0.001, respectively) while the FIM differs significantly from the FM and DM (T-HSD post hoc test, *p* ≈ 0.22 and *p* ≈ 0.53, respectively) but not from the fish-only distribution (T-HSD post hoc test, *p* ≈ 0.072). Complementary statistics are available in S1 Table of the Supporting information. Thus, while FIM still did not perform as well as the control experiments (16 degrees ≈ 8 cm arc distance), its ability to mimic the collective decision making allowed the robot to maintain the cohesion of the shoal with on average 14 degrees better than FM and 9 degrees better than DM. Moreover, the results observed are consistent over time as shown in Fig 4B depicting the average angular distance for every minute of the experiment.

The mean performance and amount of variance in the FM model is indicative of its deficiency when it with regard to its ability to be accepted and integrated with the shoal. Furthermore, such result is in direct contradiction with FM’s explicit goal, which was to head towards the densest point of the shoal and thus promote a more cohesive behavior, and could suggest that its movement patterns were too aggressive to be accepted by the shoal and contribute to its operation. DM, on the other hand, performed on average worse than the fish-only but exhibits similar variance and seemed to perturb the shoaling behavior less. Finally, FIM was the most consistent over time which could be indicative of an overall better acceptance by the shoal.

### Collective U-turns

**Fig 5:**
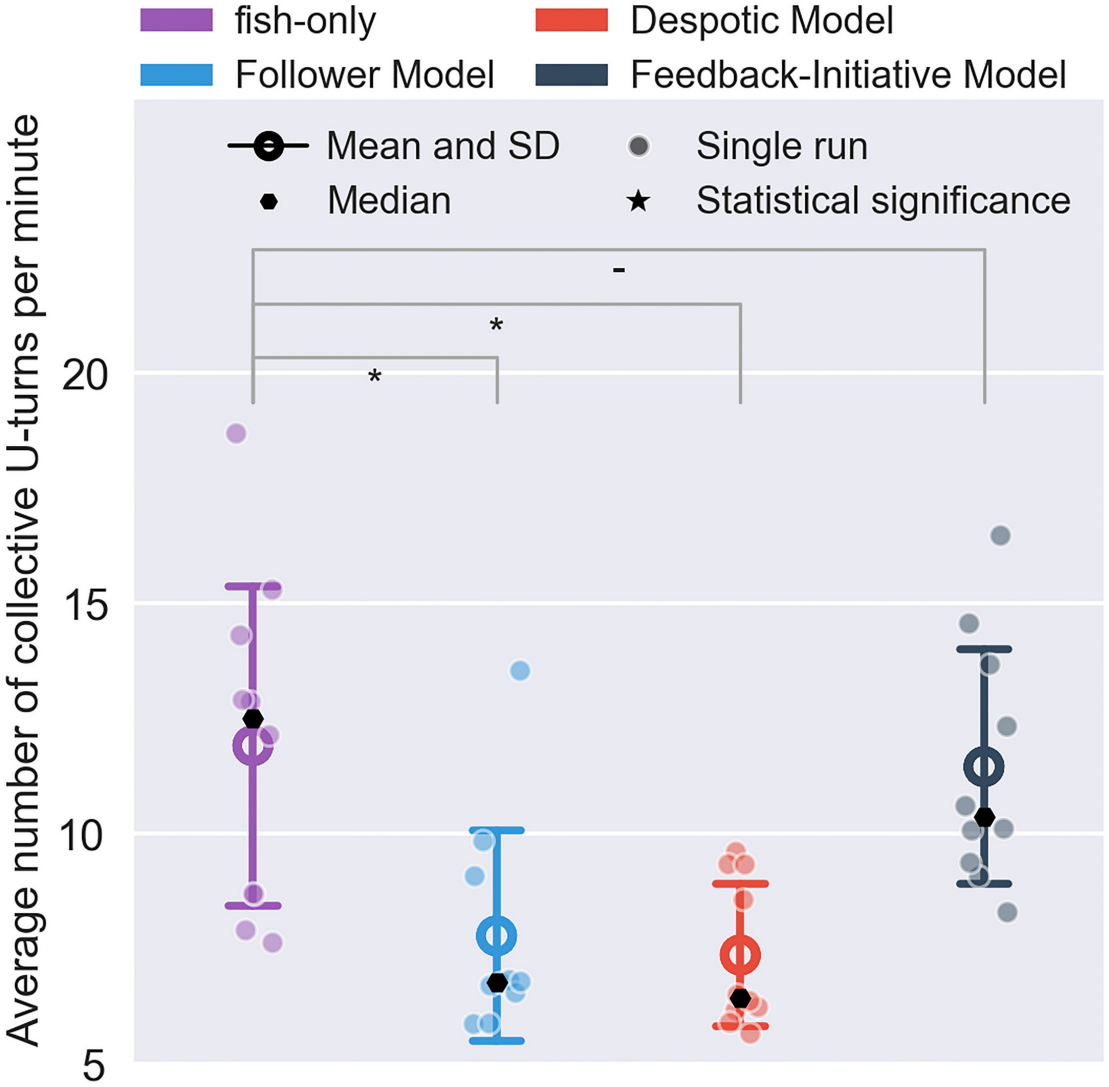
Average number of collective U-turns per minute over all replicates. Annotations of the statistical significance (Kruskal–Wallis test folowed by Tukey’s honest significant difference criterion post hoc analysis) are marked with a dash or stars. The dash corresponds *p* > 0.05, a single star to *p* < 0.05, two stars to *p* < 0.01, three stars to *p* < 0.001. and four stars to *p* < 0.0001.

The collective U-turns performed per minute (see Fig 5). (1) fish-only had median of 13.05 turns and a mean of 12.38 ± 3.71, (2) FM had median of 7.25 turns and a mean of 8.27 ± 2.30, (3) DM had median of 6.97 turns and a mean of 7.91 ± 1.60 and (4) FIM had median of 12.38 turns and a mean of 11.91 ± 2.77. These distribution differ significantly from each other (KW test, *p* < 0.001 and *χ*^2^ = 16.63). An additional post hoc T-HSD analysis showed that: FM versus fish-only had significantly different mean rank (*p* < 0.05); DM versus fish-only also showed significantly different mean rank (*p* < 0.001); FIM versus fish-only showed no significant difference (*p* > 0.99) but FIM versus FM (*p* < 0.05) and DM (*p* < 0.01) showed a significant difference (a detailed table of the post hoc analysis is available in S2 Table of the Supporting information).

These results showed that FM’s and DM’s poor performance in terms of angular distance translated in poor performance in terms of collective U-turns. While this was to be expected for the DM that was instructing the robot to move CW, FM once again appeared to disrupt the collective dynamics of the shoal. More specifically, DM’s low number of U-turns demonstrated its ability to influence the collective decision making rather than to participate in it (the shoal moves CW ≈ 65% of the time similar to [27]). Conversely, there was no significant difference between FIM and the fish-only experiments regarding the collective U-turns. This indicates that FIM had strong biomimetic capabilities due to its design, that explicitly embedded the ability to follow but also initiate direction changes.

**Fig 6:**
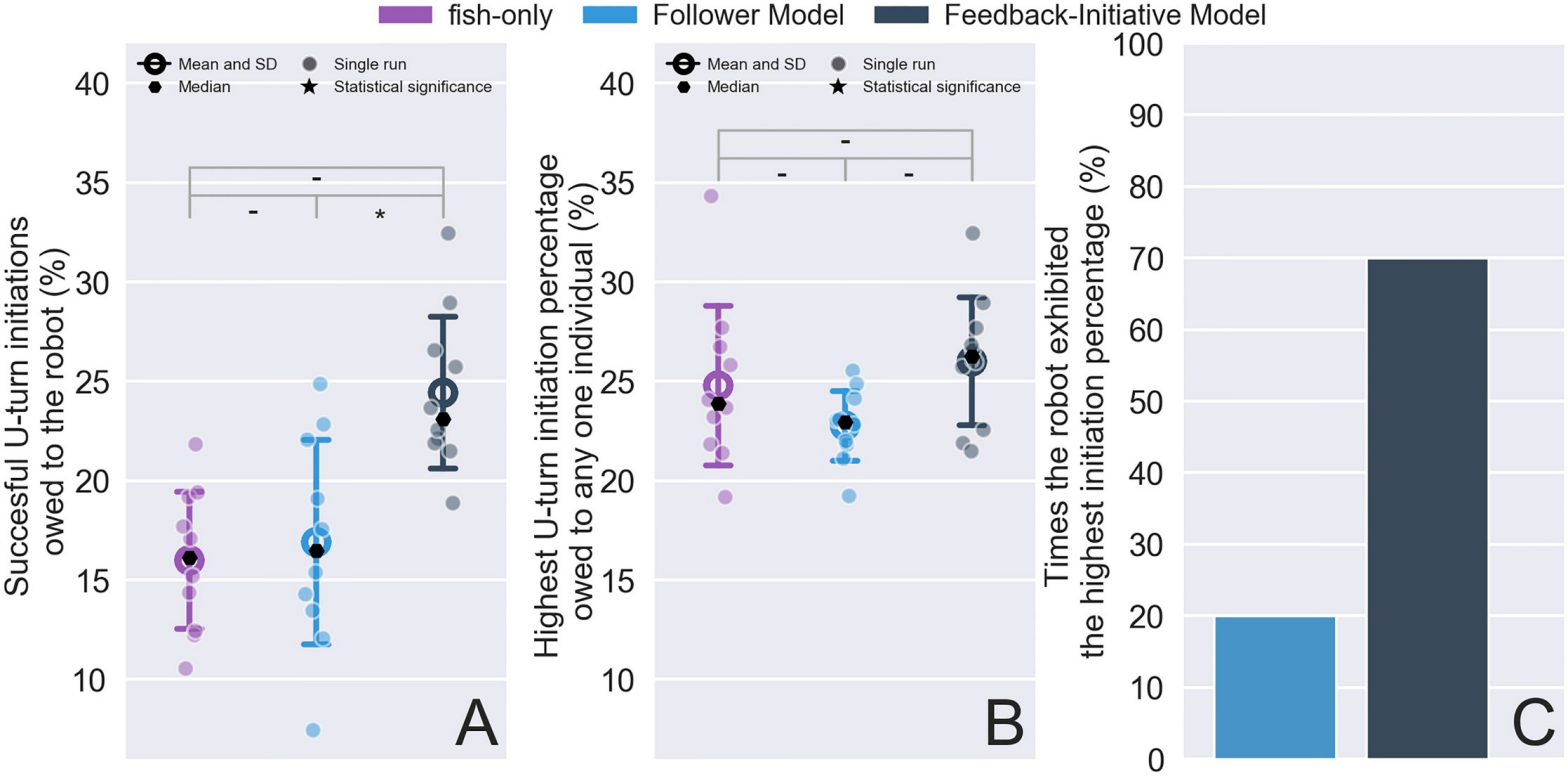
U-turn initiation success rates. (A) successful U-turns that were initiated by the robot (or a random individual in the case of fish-only), (B) highest success rate in an experiment owed to any one individual, and (C) percentage of experiments in which the robot had the highest success rate. Annotations of the statistical significance (Kruskal–Wallis test folowed by Tukey’s honest significant difference criterion post hoc analysis) are marked with a dash or stars. The dash corresponds *p* > 0.05, a single star to *p* < 0.05, two stars to *p* < 0.01, three stars to *p* < 0.001. and four stars to *p* < 0.0001.

In addition to the collective U-turns, we also investigated the success rate of the robot to initiate a collective U-turn. In Fig 6A we depict the percentage of successful U-turns that were owed to the robot’s motion, in Fig 6B we depict the highest percentage of successful U-turns exhibited by any one individual taking part in the experiment, and in Fig 6C we depict the percentage of the robot that was the most influential individual. We note that in the case of the fish-only experiments we chose one random individual and we excluded the DM experiments since the robot would never perform a U-turn. Intuitively, the above measurements can provide an estimate of the leadership characteristics of each model compared to the innate behavior of the zebrafish. Moreover, the distributions depicted in Fig 6A and 6B provide, once again, insight on the degree to which the robot might have been perturbing or naturally interacting with the living individuals.

We performed KW test for the successful U-turn initiation distribution and obtained the values of *p* < 0.05 and *χ*^2^ = 7.65. A follow-up T-HSD post hoc test revealed that fish-only does not differ significantly from the FM (*p* > 0.5), while it did indeed differ significantly from FIM (*p* < 0.5). FM also differs significantly from FIM (*p* < 0.05). While Fig 6B alone does not provide a lot of additional information (the distributions d not differ significantly KW test, *p* > 0.05), in combination with Fig 6A we notice that the robot’s U-turn initiation success rate was very similar to the distribution for the fish-only individuals with the highest success rate. In Fig 6C we quantified the latter in terms of the percentage that the robot acted as the leading individual and found than in FM experiments this corresponds to 20% and in FIM experiments to 70%. While FIM was clearly more successful in initiating a U-turn, FM’s success rate was greater than what could be expected by a following model. This is, was fact, directly linked to the densest point alternation (see Follower Model (FM)), which could very well have triggered a U-turn for the robot if the densest centroid appeared in the reverse direction. Overall, this provides evidence that the FIM was capable of producing patterns that did not perturb the collective and at the same time allowed it to have a leadership role with higher, compared to a random individual, percentage.

### Transfer entropy

**Fig 7:**
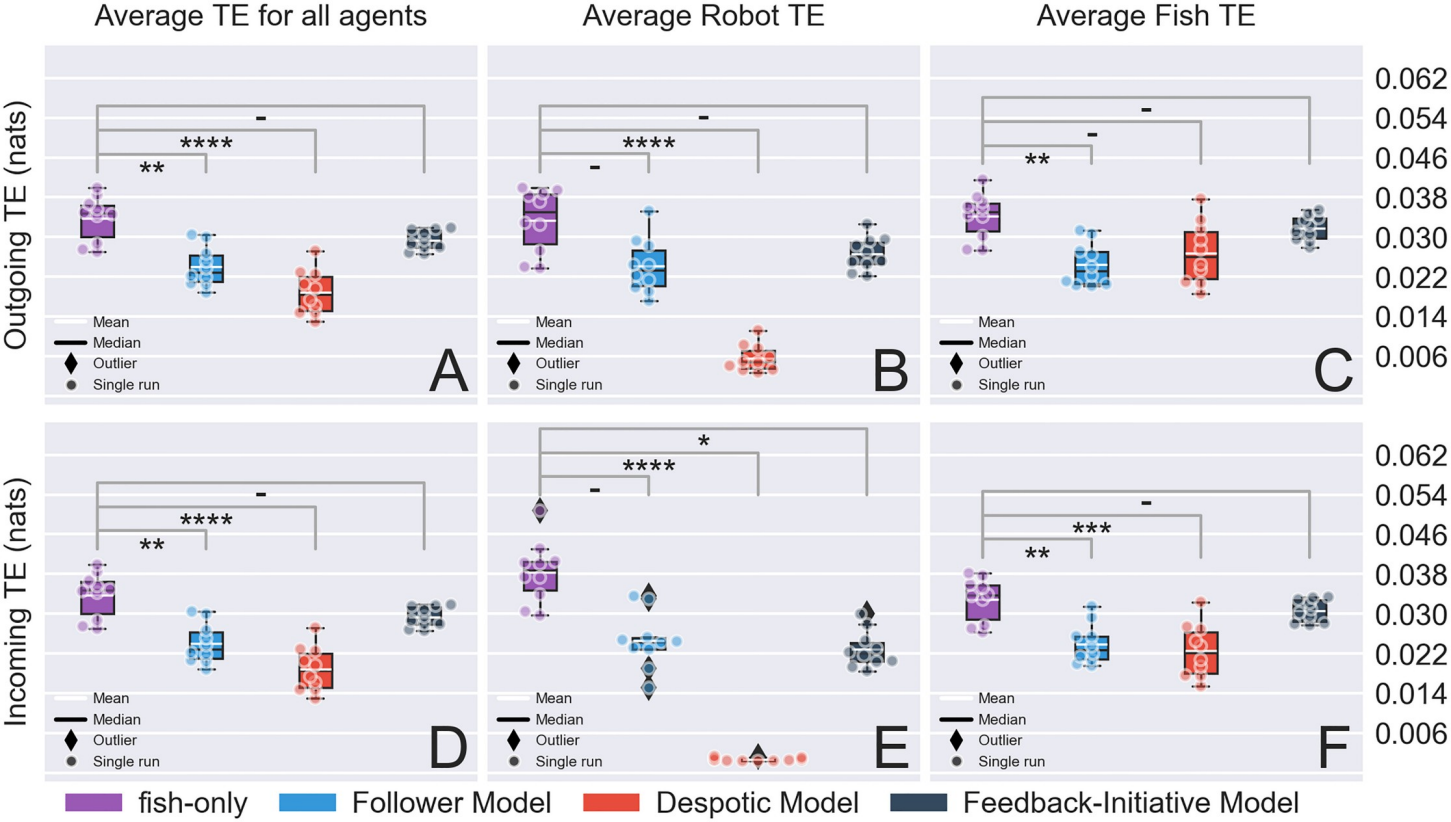
Average transfer entropy (TE) for direction time-series. The first row (i.e., Fig A, B, C) corresponds to the average outgoing TE of the shoal (i.e., amount of TE from focal towards other individuals) and the second (i.e., D, E, F) corresponds to the average incoming TE (i.e., amount of TE from other individuals towards the focal). A & D: TE for the mixed group (all individuals are considered), B & E: TE only for the robotic agent (for fish-only experiments a random fish replaces the robot) and C & F: TE only for living individuals (i.e. the robot is excluded in the computation and for fish-only experiments a random fish and excluded from the analysis). Annotations of the statistical significance (Kruskal–Wallis test followed by Tukey’s honest significant difference criterion post hoc analysis) are marked with a dash or stars. The dash corresponds *p* > 0.05, a single star to *p* < 0.05, two stars to *p* < 0.01, three stars to *p* < 0.001. and four stars to *p* < 0.0001. The complete pairwise comparisons can be found in S5, S6, S7 and S8 Tables, name, S10 Table.

We complete this section by evaluating the information propagation capabilities of each model by resorting to information theory and more specifically to the use of TE (see section Transfer entropy). In Fig 7, we measured the TE for all shoal members (fish and robots) quantifying the average influence that individuals exerted on (outgoing TE) or received from (incoming TE) the others.

First, we analyzed the average outgoing entropy for all agents (Fig 7A). Again, the distributions significantly differ from each other across the different treatments (KW test, *p* < 0.00001, and *χ*^2^ = 26.78). The fish-only condition, that showed the highest TE values, differs significantly from the FM (T-HSD post hoc test, *p* < 0.01) and DM (T-HSD post hoc test, *p* < 0.0001) but not from the FIM (T-HSD post hoc test, *p* ≈ 0.59).

The lower performance observed in the mixed groups could be partly attributed to the robot’s slow response to stimuli or the models’ lack of locomotive aspects that might play an important role in good integration (e.g., biomimetic locomotion patterns). On the other hand, it is important to note that the amount of outgoing directional information exchanged did not significantly differ in the case of FIM versus fish-only which in turn implies that the robotic lure had a considerable impact on the shoal. In fact, FIM stood out compared to the rest of the models in terms of distribution similarity, therefore, we conclude that its biomimetic decision making was indeed important when it came to propagating information within the shoal.

To highlight the role played by the robot in the shoal dynamics, we separated the average outgoing TE of the robot (Fig 7B) and the average outgoing TE of the fish (Fig 7C). The intuition behind this threefold separation (average TE for all agents, average robot TE, average fish TE) is summarized as follows: (1) an overall estimate of how each model affected directional information transfer in the shoal, (2) a quantification of the robot’s interaction with the fish and (3) an evaluation of the perturbations in information transfer between the fish due to the presence of the robot. We note that for the fish-only box-plots of Fig 7B and 7C, we chose one fish at random since no robot is used.

For the robot TE case in Fig 7B, the distributions differ significantly (KW test, *p* < 0.00001 and *χ*^2^ = 27.19). A multiple comparison of the distributions showed that: fish-only did not differ significantly from FM (T-HSD post hoc test, *p* ≈ 0.11) and FIM (T-HSD post hoc test, *p* ≈ 0.54) but differed significantly from DM (T-HSD post hoc test, *p* < 0.0001).

Then, we computed the average outgoing TE exchange only among the fish for the different conditions (Fig 7C). The KW test showed that the distributions differ significantly with each other (KW test, *p* < 0.05). The multiple comparisons post hoc test revealed that: fish-only differ significantly from FM (T-HSD post hoc test, *p* < 0.01), but not from DM (T-HSD post hoc test, *p* > 0.05) and FIM (T-HSD post hoc test, *p* ≈ 0.93). In these cases, FIM performed closely to the control experiments. We believe that this could be linked to the degree of acceptance of the robot by the society. More specifically, if the robot does not perturb the directional information propagation, or ideally contributes to it, it might have higher chances to be accepted as a conspecific. In that respect, FIM seems to be the better model out of the ones we tested.

Similarly, we computed the average incoming TE for all individuals Fig 7D, only the robot Fig 7E, and only the fish for all conditions Fig 7F. For the incoming TE for all agents, the results were identical to the one obtained for the average outgoing TE shown in Fig 7A as the amount of information exchanged is preserved within the system but distributed differently among the fish and the robot.

Concerning the average incoming TE of the robot (see Fig 7E), the distributions were significantly different, as observed for the average outgoing TE (KW test, *p* < 0.0001, *χ*^2^ = 32.21). From the complementary T-HSD post hoc analysis we obtained the values *p* ≈ 0.07, *p* < 0.0001, *p* < 0.05 for fish-only versus FM, fish-only versus DM and fish-only versus FIM, respectively. In this case, the FIM under-performed compared to FM. However, this could be expected as the FM, a purely reactive model, was constantly instructing the robot to follow the fish while the FIM could lead the robot to take an initiative that contradicted the behavior of the fish.

For the average incoming TE of only the fish (Fig 7F), we also observed a significant effect of the conditions on the average TE (KW test, *p* < 0.001). However, contrarily to the average outgoing TE, the multiple comparisons showed that fish-only significantly differs from the FM (T-HSD post hoc test, *p* < 0.01) and DM (T-HSD post hoc test, *p* < 0.001) but not from the FIM (T-HSD post hoc test, *p* ≈ 0.89). These results confirmed that the robot controlled by the FIM did not impede the transfer of information between the fish.

## Discussion

Testing theoretical hypotheses in realistic conditions is an imperative step towards understanding the collective dynamics of natural systems. However, generating specific patterns that are valuable to validate those hypotheses requires sophisticated physical systems. In the case of animal studies, and specifically the study of zebrafish’s group interactions, such systems must blend well enough in the shoal as to allow for natural and life-like interaction dynamics to emerge. Thus, apart from visual biomimetic cues, a robotic device ought to behave as close to the living creature as possible. In turn, this raises questions on the necessity of complex behavioral models in order for an artificial agent to socially interact with a high degree of integration in the group. Here, we showed that a model, that has been simplified to be implemented on a physical system, allowed the robot to establish life-like interactions with a shoal of fish through a bidirectional communication scheme.

While one could assume that a simple following model that instructs a robotic agent to move towards the fish would quite naturally succeed in “infiltrating” the shoal, we showed that our own Follower model (FM), failed to do so. Although further experiments need to be conducted to understand the underlying mechanisms that FM failed to capture, intuitively, a lure that is not attempting to interact with the living agents is less appealing to them and at times is completely disregarded (in the collective decision-making sense). Such hypotheses were already studied in related work (e.g. [26]), but, here, we were able to do so in a reproducible way, in experiments of 30 minutes, with a closed-loop interaction between the animals and the robot that was permanent during the entire experiment. The results of the previous section also showed that this passive control scheme perturbed the behavior of the fish as we observed greater mean inter-individual distance and at the same time fewer U-turns performed on the global scale. This was also validated by visual inspection of the corresponding experiment recordings. Moreover, we attempted to trace the source of this failure by using a TE metric to estimate the amount of information that is exchanged when this model is active on the robot. While such a metric can not be used to safely draw causal conclusions, the results implied that there was a significantly different trend in the information flow for this model (see Fig 7) that could explain the lack of similarity on the global scale (i.e., inter-individual distance, U-turns and successful U-turn initiation).

Another baseline, yet very informative, experiment we conducted is related to the Despotic model (DM). In contrary to the FM, we set out to test the response of the fish when the scenario is inverted, that is when the robot is disregarding their decisions concerning the direction of movement. More specifically, we aimed to test two extreme cases and observed the responses for each one. As shown in section Feedback-Initiative Model (FIM)., DM also fails to capture the interest of the living individuals for long periods of time. Conversely, the Feedback-Initiative model (FIM) managed to exhibit patterns that proved to lead to similar dynamics on a global scale. Especially the results depicted in Fig 5 concerning the collective U-turns, showed that the living individuals interact with the robot and between them in a similar manner as groups of only fish. Similarly, the TE measurements implied that the robot managed to establish stronger communication channels with the fish that could, in turn, explain the similarity of the U-turn distributions. Even more interestingly, we noticed that the robot had a leading role (i.e., a direction change of the robot was likely to propagate to the remaining group members) for the majority of the experiments and had a very similar influence to the most influential fish individual of the fish-only experiments.

In this study, we implemented different behavioural models on a robot-fish to test their ability to interact with the bio-hybrid group members. In particular, we segmented the model space in three categories spanning it; (1) passive models (FM), (2) reactive models (DM), and (3) intermediate models, like the FIM where the robot alternates between (1) and (2). The contribution of this work lies in the following two; (1) demonstrating that the robotic framework we developed is able to exhibit encoded models to study the collective behavior of small fish species, such as the one of [61] from which the FIM was inspired, and (2) the comparative study of those three fundamentally different approaches to the robot control problem and it constitutes a first towards building more complex models that can elicit even more complex responses by leveraging the findings of this study.

### Limitations

Embedded systems have come a long way in the past century and have allowed for the miniaturization of robotic systems to the point where they can interact with small animals like fish [22–24, 26]. While most of those systems can achieve similar acceleration and velocity profiles to the zebrafish themselves, they are still constrained by the physical laws. That is, such systems need to account for friction, are often bound to the trade-off of size versus controllability, typically rely on additional parts that affect water flow and in the case of multi-robot systems they need to maintain a safe distance (e.g., fish tend to cross on the y-axis but to this day there is no physical robot that can achieve this). Here, we relied on a two-part system, a miniature robot and a lure, that has already proved to be significantly biomimetic to “blend” in a shoal of fish [22]. In some cases, we found that each of the models presented required a considerable amount of manual tuning of their parameters to reach realistic motion profiles. On that end, our control procedures could benefit from the advancements in machine learning that have been shown to be able to tune such models in real-time [84]. Moreover, while the models used did not require more computational power than what was available, we expect that future experiments with multiple robots and more complex models might and, therefore, the future work needs to focus on both data-efficient algorithms for the adaptation step of the robot and to making use of modern computational systems that can deal with this intensive load.

An alternative approach that was proposed in order to address some of the physical limitations of such a robotic system was the use of animated images (e.g., a fish image) projected into the experimental arena [59]. This would in principle allow for very rapid movements without the complexity owed to the use of physical systems that can not, for example, operate in very small distances. However, while such systems offer a great alternative, in the case of zebrafish and animals in general there might be interactions that are exclusively elicited through physical contact or properties (e.g., the zebrafish will perceive changes in water flow and adapt accordingly).

In addition to the physical limitations of such a complex robotic system, we were also faced with the task of ensuring that the individuals used in this study were not acclimatized to the effects of the robot. For this purpose, we used the same protocol established and validated in our prior studies, that is, the models were encoded in a random way for sequential experiments and no fish was used twice each day. The policy enforced by the host country (Switzerland) concerning the refined and reduced usage of animals for experimentation allowed for the of only two aquariums and a small number of fish (see section Experimental procedure). Since the fish had to be returned in the two aquariums available at the time, separating the groups tested was not feasible. To address this, we randomly caught fish from the aquariums to create mixed populations with different prior experiences and account for the same robot effect.

### Future work

The evaluation of the extent to which the robot can “blend” in a group of living animals and socially interact with them is imperative in order to establish useful and informative baselines about the design, implementation and more importantly the prospects of mixed groups of animals and robots. A robotic tool capable of “convincing” its social companions that it is part of their group is likely the first step towards an ethological tool that allows for probing very specific responses from the bio-hybrid group. For this to happen, there is the need for realistic decision making models that can lead meaningful interactions, for robots agile enough to adapt to the quick responses of the fish and for robust and data-efficient algorithms that can potentially tune the models online to allow the robot to integrate in the group for long periods of time. The results conducted within this research work, imply that a highly integrated agent might be more influential and well integrated with a group of 5 zebrafish. While such a claim needs to be studied further, it becomes more obvious that to validate collective behavior models, data simulations are not sufficient as they can not model or predict its true impact on the individuals themselves, that is, there is a reality gap between simulation and physical world. Thus, the use of robotic systems becomes increasingly more important in view of more complex studies on the underlying mechanisms of natural systems. Thus far, our platform has allowed us to test scenarios, with various fish species interacting with one or more robots that could embed similar models to the one developed in this work, in order to study complex collective behavior with groups of multiple fish and robots.

## Supporting information

S1 Text Additional details on experimentation schedule and duration. (PDF)

S1 Table Post hoc analysis for the average angular distance distributions preceded by a Kruskal–Wallis test and using Tukey’s honest significant difference. (PDF)

S2 Table Post hoc analysis for the average number of collective U-turns distributions preceded by a Kruskal–Wallis test and using Tukey’s honest significant difference criterion. (PDF)

S3 Table Post hoc analysis for the U-turn initiation success rate distributions preceded by a Kruskal–Wallis test and using Tukey’s honest significant difference criterion. (PDF)

S4 Table Post hoc analysis for the highest U-turn initiation success rate by any one individual distributions preceded by a Kruskal–Wallis test and using Tukey’s honest significant difference criterion. (PDF)

S5 Table Post hoc analysis for the average outgoing transfer entropy distributions preceded by a Kruskal–Wallis test and using Tukey’s honest significant difference criterion. Average outgoing TE including all agents.
(PDF)

S6 Table Post hoc analysis for the average outgoing transfer entropy distributions preceded by a Kruskal–Wallis test and using Tukey’s honest significant difference criterion. Average outgoing TE only for the robot’s contribution.
(PDF)

S7 Table Post hoc analysis for the average outgoing transfer entropy distributions preceded by a Kruskal–Wallis test and using Tukey’s honest significant difference criterion. Average outgoing TE only for the fish contribution.
(PDF)

S8 Table Post hoc analysis for the average incoming transfer entropy distributions preceded by a Kruskal–Wallis test and using Tukey’s honest significant difference criterion. Average incoming TE including all agents (identical to outgoing case for all agents).
(PDF)

S9 Table Post hoc analysis for the average incoming transfer entropy distributions preceded by a Kruskal–Wallis test and using Tukey’s honest significant difference criterion. Average incoming TE only for the robot’s contribution.
(PDF)

S10 Table Post hoc analysis for the average incoming transfer entropy distributions preceded by a Kruskal–Wallis test and using Tukey’s honest significant difference criterion. Average outgoing TE only for the fish contribution.
(PDF)

S1 Fig Duration of consecutive movement towards a direction (across all 10 replicates). Fish-only case.
(TIF)

S2 Fig Duration of consecutive movement towards a direction (across all 10 replicates). Fish-only case where a random individual is excluded.
(TIF)

S3 Fig Duration of consecutive movement towards a direction (across all 10 replicates). Follower model.
(TIF)

S4 Fig Duration of consecutive movement towards a direction (across all 10 replicates). Follower model with the robot excluded from the analysis.
(TIF)

S5 Fig Duration of consecutive movement towards a direction (across all 10 replicates). Despotic model.
(TIF)

S6 Fig Duration of consecutive movement towards a direction (across all 10 replicates). Despotic model with the robot excluded from the analysis.
(TIF)

S7 Fig Duration of consecutive movement towards a direction (across all 10 replicates). Feedback-Initiative model.
(TIF)

S8 Fig Duration of consecutive movement towards a direction (across all 10 replicates). Feedback-Initiative model with the robot excluded from the analysis.
(TIF)

S1 Video Recordings (segments) of the models in action. A short video depicting each model’s resulting dynamics for a duration of 60 seconds. The full videos are available upon request due to the large file size.
(MP4)
