# Supplementary material for: Bidirectional interactions facilitate the integration of a robot into a shoal of zebrafish Danio rerio
Source: PLoS One. 2019 Aug 20;14(8):e0220559. doi: 10.1371/journal.pone.0220559 (PMC6701756; doi:10.1371/journal.pone.0220559)
Supplement: S1 Text — (PDF) [file pone.0220559.s001.pdf]

The experiments were conducted between the 30th of April and 5th of June 2018 at the premises of École Polytechnique Fédérale de Lausanne. The earliest recorded experiment was conducted at 9:30 and the latest at 18:30. During the experimentation time, the zebrafish were housed with a 14 to 10 day to night ratio.
