# Supplementary material for: Bidirectional interactions facilitate the integration of a robot into a shoal of zebrafish Danio rerio
Source: PLoS One. 2019 Aug 20;14(8):e0220559. doi: 10.1371/journal.pone.0220559 (PMC6701756; doi:10.1371/journal.pone.0220559)
Supplement: S1 Table — (PDF) [file pone.0220559.s002.pdf]

**Model**	**Model**	**Lower CI**	**Estimate**	**Upper CI**	**p-value**
fish-only	Follower	-36.1312	-22.7000	-9.2688	0.0001
fish-only	Despotic	-33.2312	-19.8000	-6.3688	0.0009
fish-only	Feedback-Initiative	-26.1312	-12.7000	0.7312	0.0717
Follower	Despotic	-10.5312	2.9000	16.3312	0.9453
Follower	Feedback-Initiative	-3.4312	10.0000	23.4312	0.2226
Feedback-Initiative	Despotic	-6.3312	7.1000	20.5312	0.5258

CI stands for confidence interval.
