# Supplementary material for: Bidirectional interactions facilitate the integration of a robot into a shoal of zebrafish Danio rerio
Source: PLoS One. 2019 Aug 20;14(8):e0220559. doi: 10.1371/journal.pone.0220559 (PMC6701756; doi:10.1371/journal.pone.0220559)
Supplement: S2 Table — (PDF) [file pone.0220559.s003.pdf]

| **Model** | **Model** | **Lower CI** | **Estimate** | **Upper CI** | **p-value** |
|---|---|---|---|---|---|
| fish-only | Follower | 0.2707 | 13.7000 | 27.1293 | 0.0435 |
| fish-only | Despotic | 2.2707 | 15.7000 | 29.1293 | 0.0142 |
| fish-only | Feedback-Initiative | -14.0293 | -0.6000 | 12.8293 | 0.9995 |
| Follower | Despotic | 11.4293 | 2.0000 | 15.4293 | 0.9810 |
| Follower | Feedback-Initiative | -27.7293 | -14.3000 | -0.8707 | 0.0316 |
| Feedback-Initiative | Despotic | -29.7293 | -16.3000 | -2.8707 | 0.0098 |

CI stands for confidence interval.
