# Supplementary material for: Bidirectional interactions facilitate the integration of a robot into a shoal of zebrafish Danio rerio
Source: PLoS One. 2019 Aug 20;14(8):e0220559. doi: 10.1371/journal.pone.0220559 (PMC6701756; doi:10.1371/journal.pone.0220559)
Supplement: S3 Table — (PDF) [file pone.0220559.s004.pdf]

**Model**	**Model**	**Lower CI**	**Estimate**	**Upper CI**	**p-value**
fish-only	Follower	-5.0272	4.2000	13.4272	0.5347
fish-only	Feedback-Initiative	-15.8272	-6.6000	2.6272	0.2143
Follower	Feedback-Initiative	-20.0272	-10.8000	-1.5728	0.0168

CI stands for confidence interval.
