# Supplementary material for: Bidirectional interactions facilitate the integration of a robot into a shoal of zebrafish Danio rerio
Source: PLoS One. 2019 Aug 20;14(8):e0220559. doi: 10.1371/journal.pone.0220559 (PMC6701756; doi:10.1371/journal.pone.0220559)
Supplement: S4 Table — (PDF) [file pone.0220559.s005.pdf]

Model	Model	Lower CI	Estimate	Upper CI	p-value
fish-only	Follower	-4.3272	4.9000	14.1272	0.4269
fish-only	Feedback-Initiative	-12.9272	-3.7000	5.5272	0.6151
Follower	Feedback-Initiative	-17.8272	-8.6000	0.6272	0.0738

CI stands for confidence interval.
