# Supplementary material for: Bidirectional interactions facilitate the integration of a robot into a shoal of zebrafish Danio rerio
Source: PLoS One. 2019 Aug 20;14(8):e0220559. doi: 10.1371/journal.pone.0220559 (PMC6701756; doi:10.1371/journal.pone.0220559)
Supplement: S5 Table — Average outgoing TE including all agents. (PDF) [file pone.0220559.s006.pdf]

Model	Model	Lower CI	Estimate	Upper CI	p-value
fish-only	Follower	3.9688	17.4000	30.8312	0.0048
fish-only	Despotic	11.3688	24.8000	38.2312	0.0000
fish-only	Feedback-Initiative	-6.8312	6.6000	20.0312	0.5870
Follower	Despotic	-6.0312	7.4000	20.8312	0.4896
Follower	Feedback-Initiative	-24.2312	-10.8000	2.6312	0.1644
Feedback-Initiative	Despotic	-31.6312	-18.2000	-4.7688	0.0028

CI stands for confidence interval.
