# Supplementary material for: Bidirectional interactions facilitate the integration of a robot into a shoal of zebrafish Danio rerio
Source: PLoS One. 2019 Aug 20;14(8):e0220559. doi: 10.1371/journal.pone.0220559 (PMC6701756; doi:10.1371/journal.pone.0220559)
Supplement: S6 Table — Average outgoing TE only for the robot’s contribution. (PDF) [file pone.0220559.s007.pdf]

Model	Model	Lower CI	Estimate	Upper CI	p-value
fish-only	Follower	-1.5312	11.9000	25.3312	0.1036
fish-only	Despotic	12.8688	26.3000	39.7312	0.0000
fish-only	Feedback-Initiative	-6.4312	7.0000	20.4312	0.5380
Follower	Despotic	0.9688	14.4000	27.8312	0.0300
Follower	Feedback-Initiative	-18.3312	-4.9000	8.5312	0.7848
Feedback-Initiative	Despotic	-32.7312	-19.3000	-5.8688	0.0013

CI stands for confidence interval.
