# Supplementary material for: Bidirectional interactions facilitate the integration of a robot into a shoal of zebrafish Danio rerio
Source: PLoS One. 2019 Aug 20;14(8):e0220559. doi: 10.1371/journal.pone.0220559 (PMC6701756; doi:10.1371/journal.pone.0220559)
Supplement: S7 Table — Average outgoing TE only for the fish contribution. (PDF) [file pone.0220559.s008.pdf]

Model	Model	Lower CI	Estimate	Upper CI	p-value
fish-only	Follower	5.0688	18.5000	31.9312	0.0023
fish-only	Despotic	-0.3312	13.1000	26.5312	0.0590
fish-only	Feedback-Initiative	-10.2312	3.2000	16.6312	0.9283
Follower	Despotic	-18.8312	-5.4000	8.0312	0.7301
Follower	Feedback-Initiative	-28.7312	-15.3000	-1.8688	0.0180
Feedback-Initiative	Despotic	-23.3312	-9.9000	3.5312	0.2307

CI stands for confidence interval.
