# Supplementary material for: Bidirectional interactions facilitate the integration of a robot into a shoal of zebrafish Danio rerio
Source: PLoS One. 2019 Aug 20;14(8):e0220559. doi: 10.1371/journal.pone.0220559 (PMC6701756; doi:10.1371/journal.pone.0220559)
Supplement: S9 Table — Average incoming TE only for the robot’s contribution. (PDF) [file pone.0220559.s010.pdf]

**Model**	**Model**	**Lower CI**	**Estimate**	**Upper CI**	**p-value**
fish-only	Follower	-0.7312	12.7000	26.1312	0.0717
fish-only	Despotic	16.0688	29.5000	42.9312	0.0000
fish-only	Feedback-Initiative	2.3688	15.8000	29.2312	0.0134
Follower	Despotic	3.3688	16.8000	30.2312	0.0072
Follower	Feedback-Initiative	-10.3312	3.1000	16.5312	0.9342
Feedback-Initiative	Despotic	-27.1312	-13.7000	-0.2688	0.0436

CI stands for confidence interval.
