# Supplementary material for: Bidirectional interactions facilitate the integration of a robot into a shoal of zebrafish Danio rerio
Source: PLoS One. 2019 Aug 20;14(8):e0220559. doi: 10.1371/journal.pone.0220559 (PMC6701756; doi:10.1371/journal.pone.0220559)
Supplement: S10 Table — Average outgoing TE only for the fish contribution. (PDF) [file pone.0220559.s011.pdf]

Model	Model	Lower CI	Estimate	Upper CI	p-value
fish-only	Follower	4.6688	18.1000	31.5312	0.0030
fish-only	Despotic	6.6688	20.1000	33.5312	0.0007
fish-only	Feedback-Initiative	-9.6312	3.8000	17.2312	0.8864
Follower	Despotic	-11.4312	2.0000	15.4312	0.9810
Follower	Feedback-Initiative	-27.7312	-14.3000	-0.8688	0.0317
Feedback-Initiative	Despotic	-29.7312	-16.3000	-2.8688	0.0099

CI stands for confidence interval.
