# Supplementary figures and images for: Bidirectional interactions facilitate the integration of a robot into a shoal of zebrafish Danio rerio
Source: PLoS One. 2019 Aug 20;14(8):e0220559. doi: 10.1371/journal.pone.0220559 (PMC6701756; doi:10.1371/journal.pone.0220559)

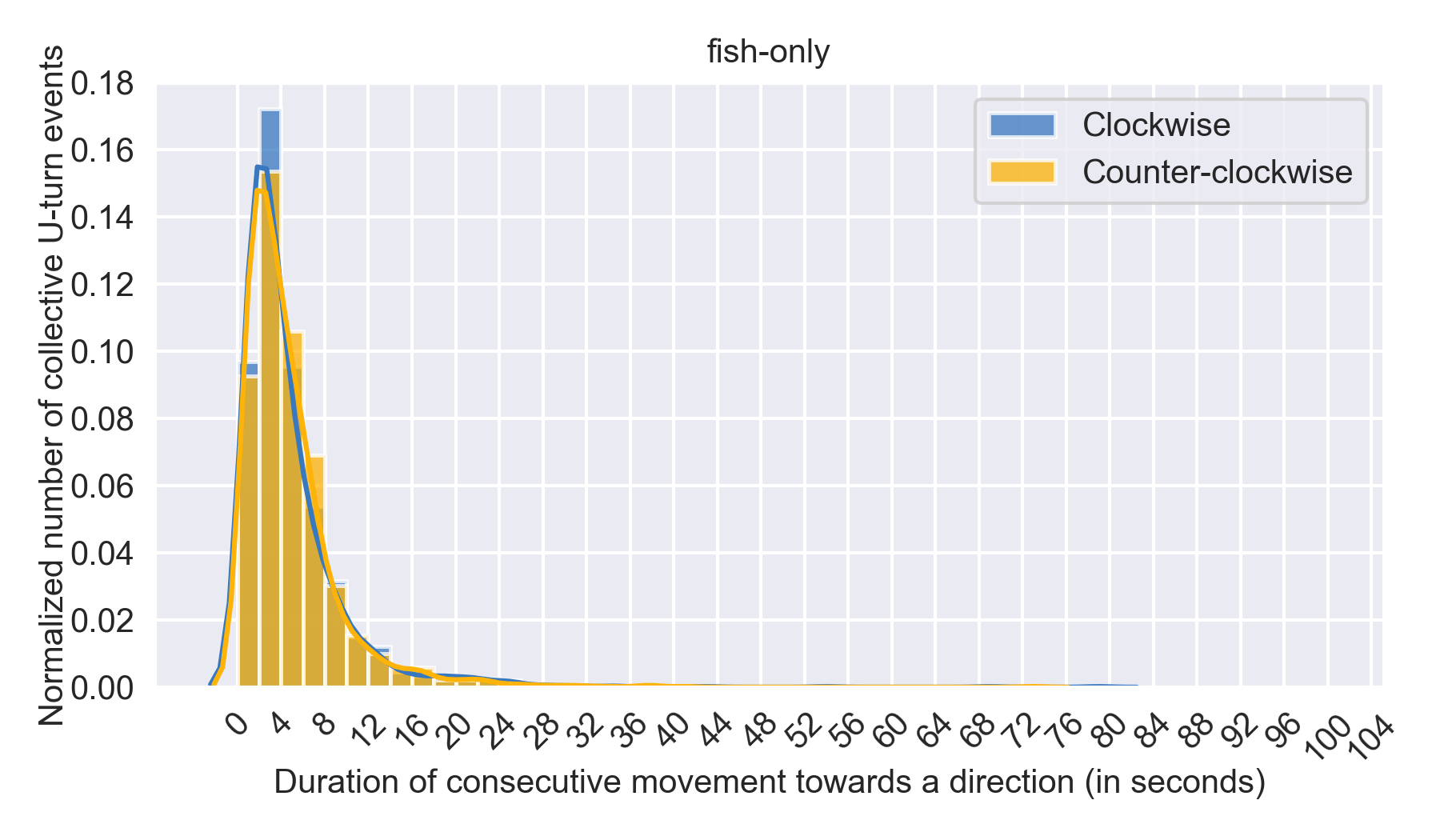

Supplement: S1 Fig — Fish-only case. (TIF) [file pone.0220559.s012.tif]

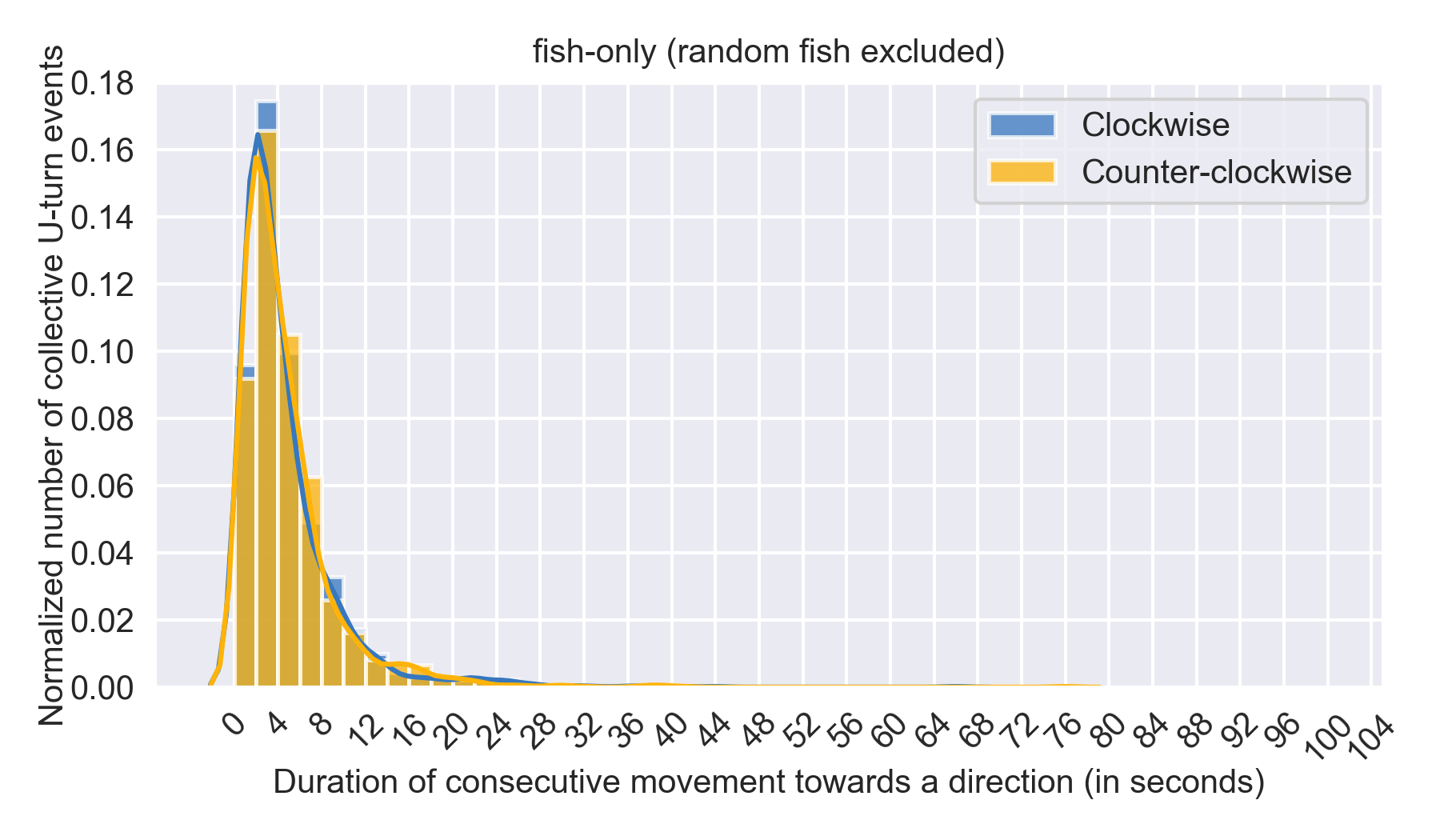

Supplement: S2 Fig — Fish-only case where a random individual is excluded. (TIF) [file pone.0220559.s013.tif]

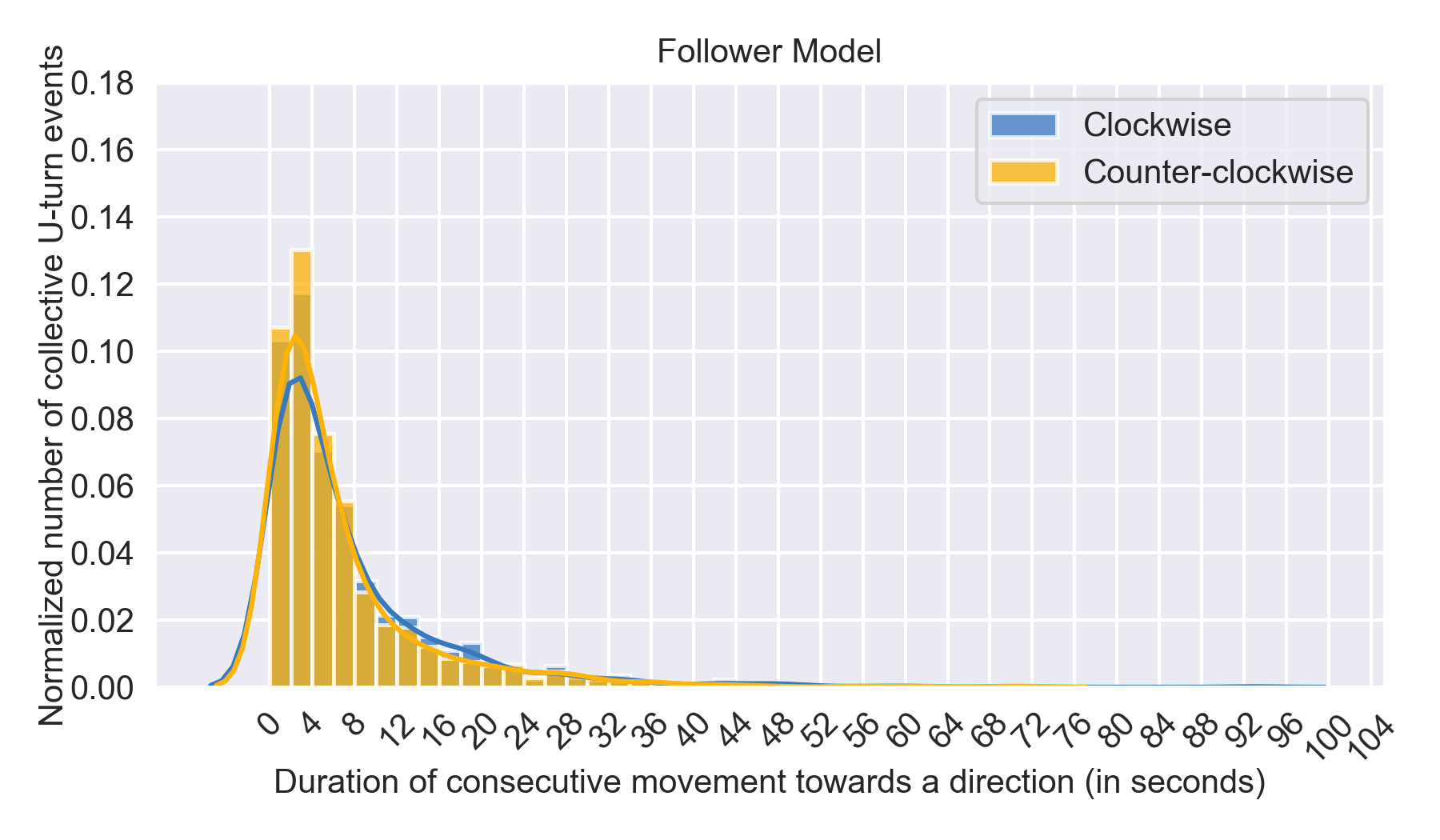

Supplement: S3 Fig — Follower model. (TIF) [file pone.0220559.s014.tif]

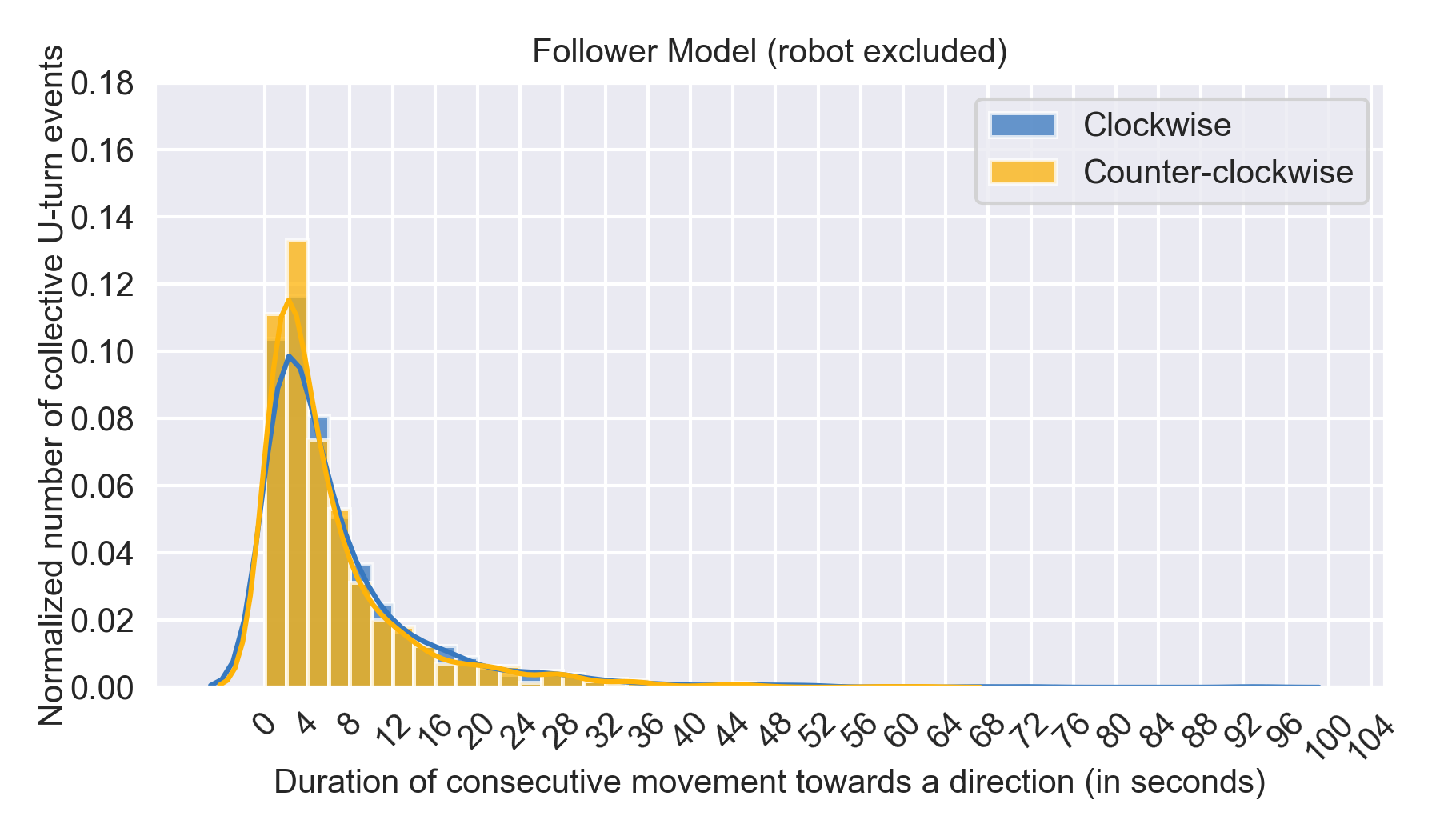

Supplement: S4 Fig — Follower model with the robot excluded from the analysis. (TIF) [file pone.0220559.s015.tif]

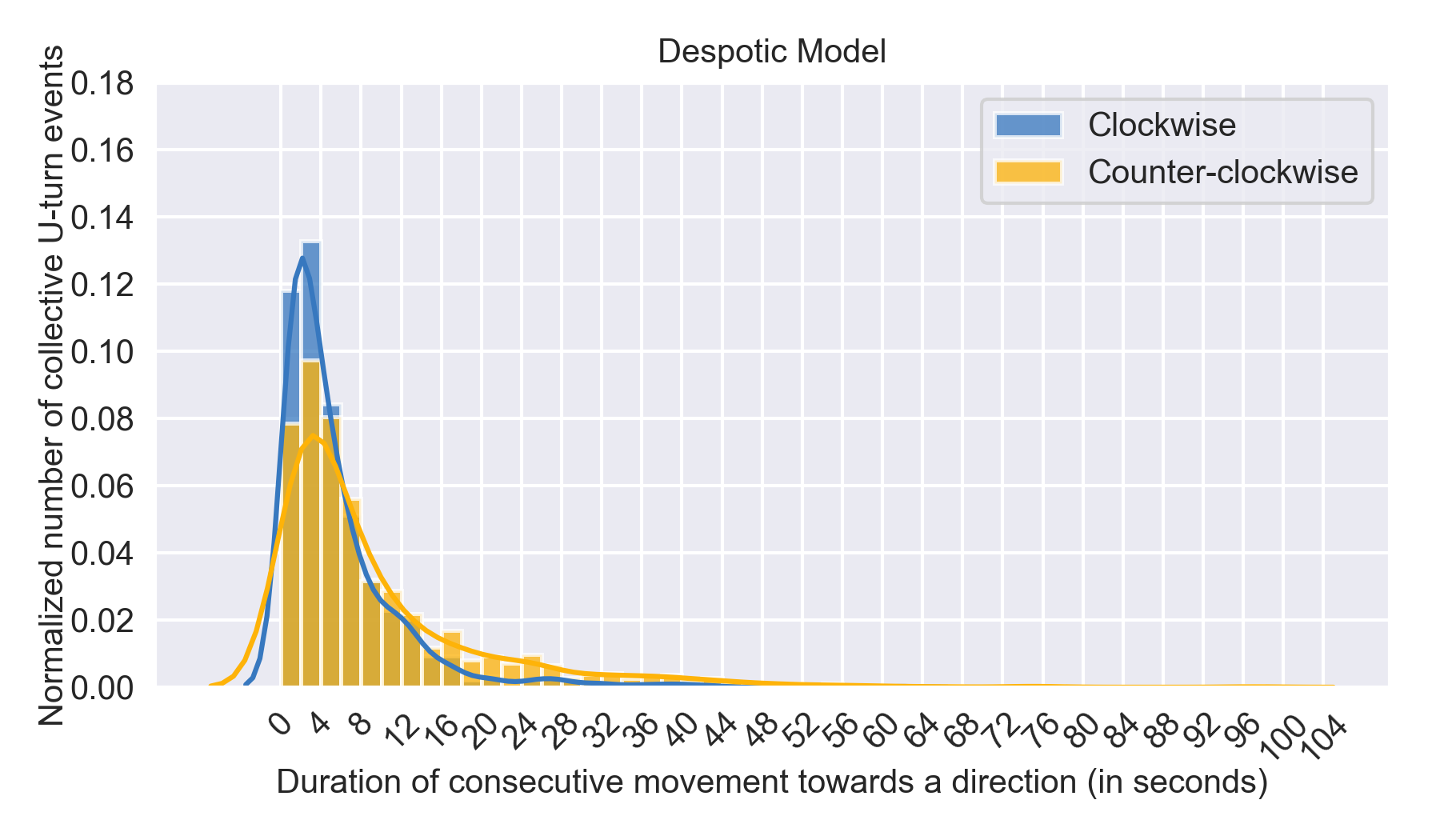

Supplement: S5 Fig — Despotic model. (TIF) [file pone.0220559.s016.tif]

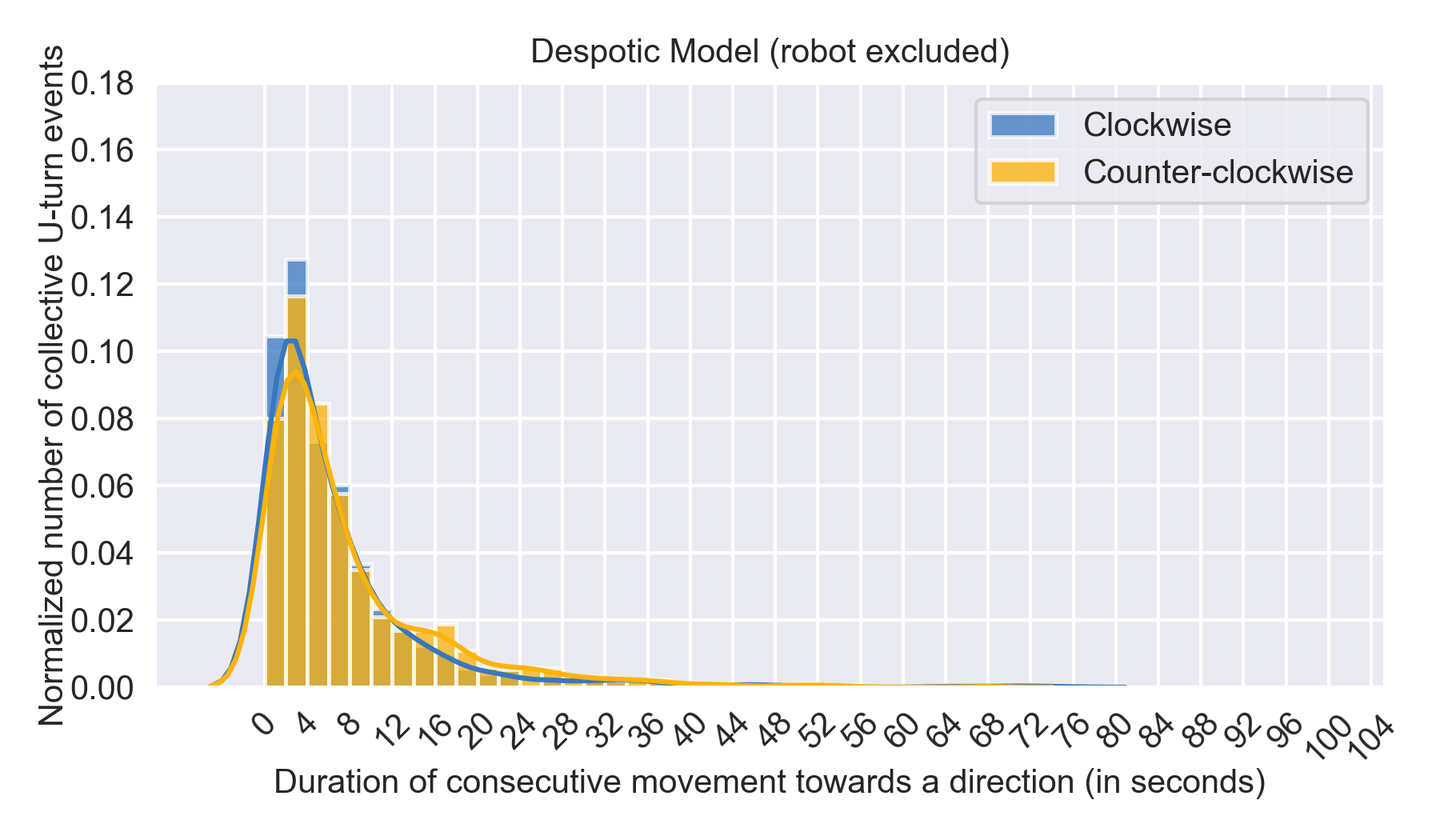

Supplement: S6 Fig — Despotic model with the robot excluded from the analysis. (TIF) [file pone.0220559.s017.tif]

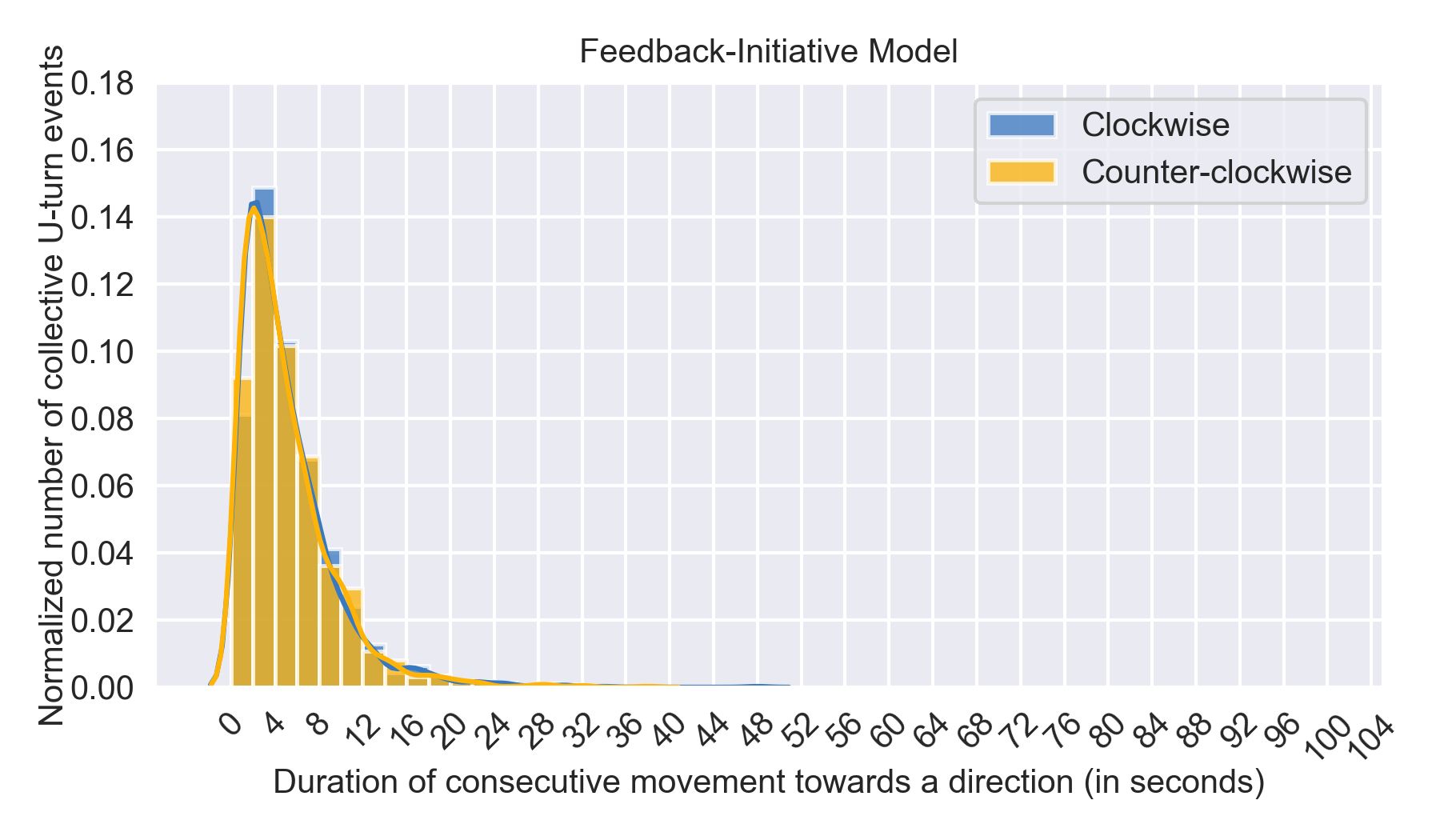

Supplement: S7 Fig — Feedback-Initiative model. (TIF) [file pone.0220559.s018.tif]

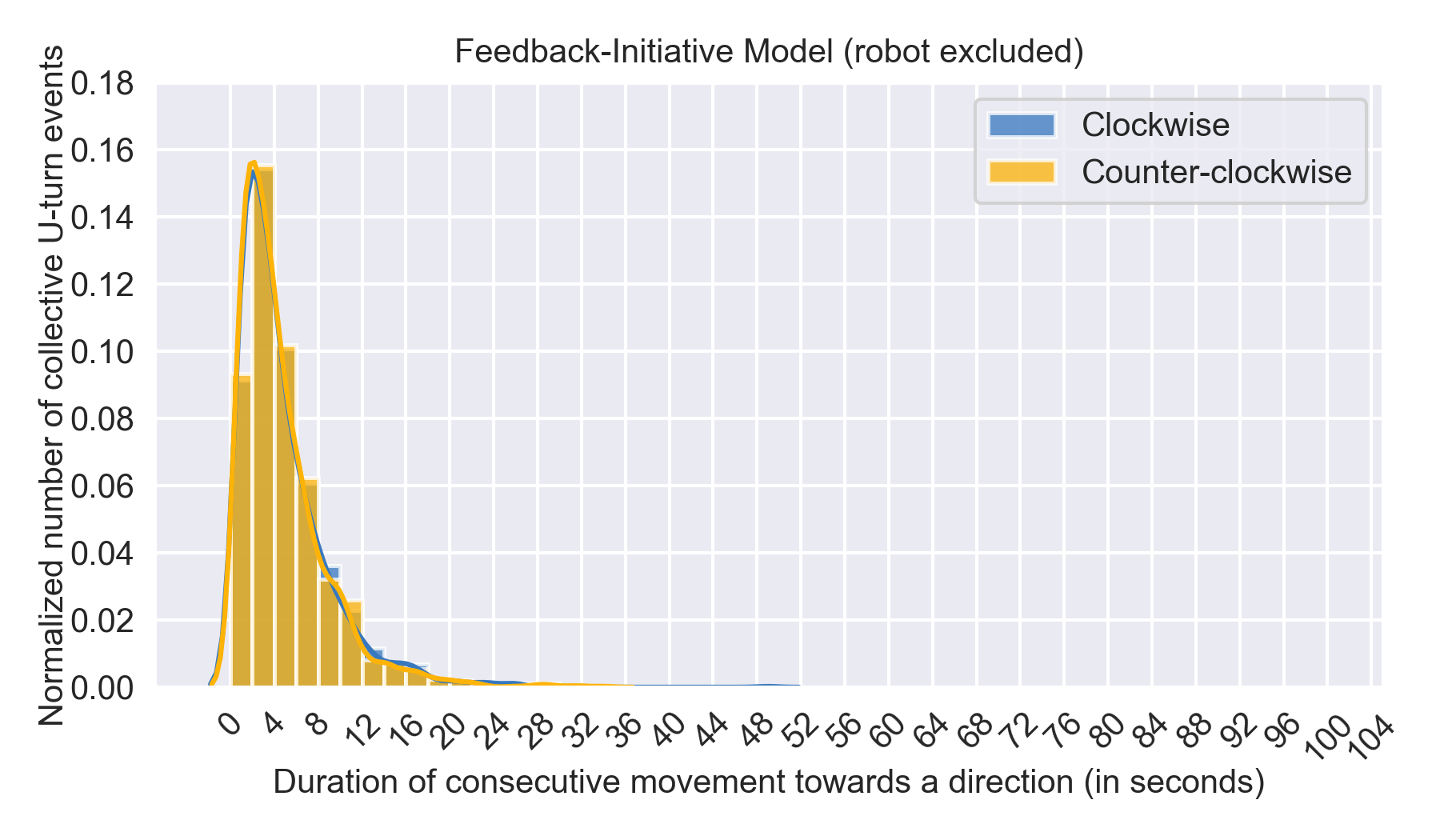

Supplement: S8 Fig — Feedback-Initiative model with the robot excluded from the analysis. (TIF) [file pone.0220559.s019.tif]
